# A Review: High-Precision Angle Measurement Technologies

**DOI:** 10.3390/s24061755

**Published:** 2024-03-08

**Authors:** Shengtong Wang, Rui Ma, Feifan Cao, Linbin Luo, Xinghui Li

**Affiliations:** 1 Tsinghua Shenzhen International Graduate School, Tsinghua University, Shenzhen 518055, China; wct21@mails.tsinghua.edu.cn (S.W.); marui@sz.tsinghua.edu.cn (R.M.); cff22@mails.tsinghua.edu.cn (F.C.); llb21@mails.tsinghua.edu.cn (L.L.); 2 Tsinghua-Berkeley Shenzhen Institute, Tsinghua University, Shenzhen 518055, China

**Keywords:** angle measurement, single-axis, multi-axis, autocollimation, artificial intelligence assistance

## Abstract

Angle measurement is an essential component of precision measurement and serves as a crucial prerequisite for high-end manufacturing. It guides the implementation of precision manufacturing and assembly. The current angle measurement methods mainly focus on multiple axes, high precision, and large measurement ranges. This article introduces the technology of angle measurement from the perspectives of single-axis and multi-axis measurement schemes. Firstly, the single-axis measurement scheme is primarily achieved through optical methods, such as encoder discs that measure energy changes and interferometric phase changes, as well as mechanical, electromagnetic, and inertial angle measurement methods, among which interferometric methods offer the highest accuracy, with high cost, and encoder discs provide the largest measurement range with an ordinary price. Secondly, in the multi-axis measurement scheme, autocollimation instruments, including plane mirrors, gratings, and self-designed targets, are the main options. Although grating encoders can achieve three degrees of freedom in angle measurement with an ordinary price, they are limited in terms of measurement range and sensitivity compared to self-designed targets. Lastly, artificial intelligence assistance precision measurement is increasingly being embraced due to significant advancements in computer performance, making it more convenient to identify the relationship between measured values and detection values. In conclusion, angle measurement plays a crucial role in precision manufacturing, and the evolving and improving technologies provide the manufacturing industry with greater choices. The purpose of this review is to help readers quickly find more suitable technical solutions according to current application requirements, such as single/multiple axes, accuracy level, measuring range, budget, etc.

## 1. Introduction

The current review of angle measurement is mainly based on the classification of measurement principles, which is not suitable for readers who are eager to find suitable angle measurement methods. Therefore, this paper mainly reviews from the perspective of application classification. The main purpose is to classify based on the current application occasions, needs, etc., to help readers quickly locate the solutions they need. This article does not discuss angles in geography and astronomy, but mainly discusses angle issues in posture changes such as mechanical motion.

As an important measurement discipline, angle measurement has run through different stages of human history, and its development origins can be traced back to ancient civilizations. The concept of angles originated in ancient Mesopotamia, specifically the ancient Babylonian civilization. The Babylonians discovered in astronomical observations that the distance the sun passes through the sky on the spring and autumn equinoxes, that is, the path that bisects the entire sky, is approximately equivalent to the length of 180 solar diameters. This phenomenon inspired the Babylonians to determine how the circumference of a complete circle should be divided, thus giving rise to the concept of the angle system. The ancient Egyptians and Mesopotamians used simple geometric principles and astronomical observation methods to measure angles, such as using sundials to measure time, constellations for navigation, etc., and tools such as square rulers and levels to measure the verticality and level of walls. These tools laid the foundation for the development of later angle measurement methods. The contribution of the ancient Greeks in the field of angle measurement cannot be ignored. Euclid’s geometric axioms laid the foundation for angle measurement. With the advancement of science and technology, angle measurement has gradually evolved into a systematic science. From the end of the 18th century to the beginning of the 19th century, with the advent of the Industrial Revolution, angle measurement methods were further developed. Engineers and scientists began to design and manufacture various precision instruments to measure angles, such as goniometers, indexing disks, etc. These instruments achieve more precise angle measurements by measuring the position and direction of rotating parts, and are widely used in manufacturing, aerospace, and other fields. The 20th century was a period of rapid development of angle measurement technology. Generally speaking, modern angle measurement methods are divided into four parts according to their principle: Photoelectric angle measurement (measuring the position and direction of a light beam through a photoelectric sensor achieves angle measurement, which is commonly used in engineering measurement and navigation systems); laser interference angle measurement (using the principle of laser interference measures angles, it has the characteristics of high precision and high speed and is widely used in manufacturing and scientific research fields); electromagnetic angle measurement (using an electromagnetic field sensor measures the direction of the magnetic field to determine the orientation and angle of an object, which is often used for electromagnetic angle encoder measurements); and inertial navigation systems (use inertial components such as gyroscopes and accelerometers to measure the rotation angle and acceleration of objects, and are widely used in robotics, industrial automation, and other fields).

In general, the development history of angle measurement technology has experienced the evolution from ancient simple geometric measurement methods to modern high-precision, multiple-axis, and large-range advanced technology. At the same time, the application of artificial intelligence and big data analysis also provides new possibilities for the further improvement of angle measurement technology. During this process, many important events and inventions played a key role in the development of angle measurement technology and made important contributions to the development and progress of human society.

Angle measurement is an important component of ultra-high-precision position and attitude measurement [1,2]. Angle measurement plays a crucial role in various fields such as civil production, industrial applications, and military operations. In the field of scientific research, it specifically includes straightness calibration [3], photon energy detection [4], grating pitch deviation measurement [5], critical dimension detection of lithography machine chips, and the splicing of space telescope lenses. According to the number of axes, high-precision angle measurement methods are divided into two types: single-axis and multi-axis. Single-axis measurement can achieve high-precision and large-scale measurement. Multi-axis measurement can achieve high-precision, multi-degree-of-freedom, and large-scale measurement simultaneously, which is the current development trend of angle measurement.

In the aspect of high-precision single-axis angle measurement, according to the working principle, it can be divided into optical methods, mechanical methods, electromagnetic methods, and inertial methods. Mechanical methods for measuring angles include multi-tooth indexing discs and tilt angle types. Electromagnetic methods include circular magnetic grating angle measurement, non/wire-wound potentiometers, micro/induction synchronizers, rotary transformers, multi-stage angle sensing motors, time gratings, etc. Inertial methods mainly include gyroscopes and accelerometers. Among all methods, optical methods are non-contact, with high accuracy and sensitivity, and are the most widely used. Optical methods include a non-interference method and interferometric method, where the non-interference method is based on energy and the interferometric method is based on phase. Interference methods include the optical indexing head method, the polyhedral prism method, the photoelectric encoder method, the circular grating method, the optical internal reflection method, the ring laser method, and other methods that are commonly used for large angle measurements, but their angle measurement accuracy depends on the stability and detection accuracy of the optoelectronic device itself. Phase-based methods such as Moiré fringes, parallel interferograms, optical frequency combs (OFCs), laser interferometry, homodyne interferometry, and heterodyne interferometry typically have high measurement accuracy and stability. The optical path of the new optical angle measurement method based on total reflection heterodyne interferometry [6] is simple, compact, and easy to align, which can achieve high resolution and a large measurement range. Measuring the absolute angle of dispersion interference using an optical frequency comb can fully utilize the quantity of dispersion interference measurement and achieve a wide range of absolute angle measurement [7,8,9]. The laser interferometry measurement method is relatively accurate, but its equipment is expensive and complex. Compared with homodyne interference, heterodyne interference has higher stability and accuracy [10].

In the aspect of high-precision multi-axis angle measurement, the main method is the autocollimator method, which uses the orientation of the reflected beam from the target for pose calculation. According to the target, high-precision multi-axis angle measurement can be divided into planar mirror, grating, and self-designed target measurement. A flat mirror, as a reflector, is used to reflect the laser and check the position of the light spot through a single PSD/CCD/QPD with high response speed. Flat mirrors are usually unable to measure roll angles, but they have the advantage of low cost. Based on autocollimators with grating to measure angles, absolute pose measurement can be achieved by measuring the angle through the change in the diffraction direction of the reflected grating [11]. Calibration is usually required when measuring the angle based on a self-designed target surface [12,13]. High-precision angle measurement can also integrate machine learning methods, using artificial intelligence to learn the changes in physical phenomena that vary with the measurement results during the measurement process, thereby automatically outputting angle measurement results, greatly improving measurement efficiency. However, the accuracy of angle measurement still needs to be further improved.

Overall, current measurement schemes are often not limited to a single measurement scheme and generally require multiple devices to work together [14]. Single-device multi-degree-of-freedom (DOF) measurement [15,16,17,18,19,20,21] or multi-device collaborative work [22] is currently a research hotspot, which can achieve the complete construction of position and orientation information [23]. At the same time, modular design can be better integrated into other devices to achieve angle detection and correction faster.

**Figure 1:**
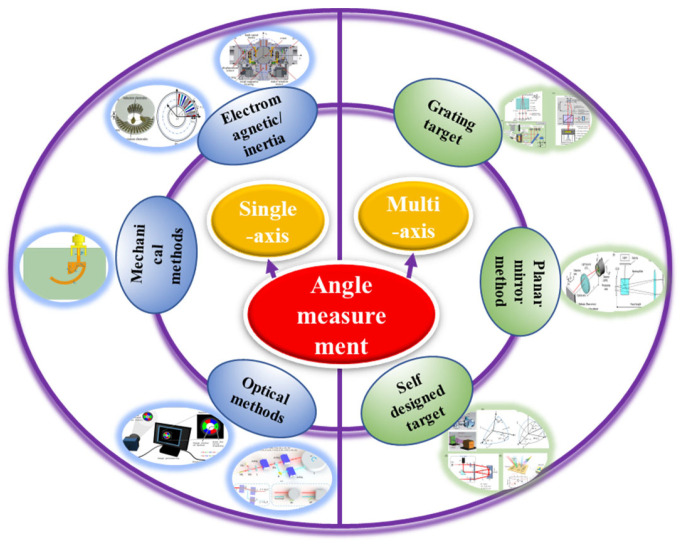
Classification logical structure of angle measurement methods.

Section 1 introduces the application background and classification of high-precision angle measurement. Section 2 and Section 3, respectively, elucidate single-axis and multi-axis angle measurement methods and the current application status. Section 4 analyzes the current problems and the future development trends. The classification logical structure of angle measurement methods is shown in Figure 1.

## 2. High-Precision with Single Axis

### 2.1. Optical Methods

The optical scheme is mainly divided into interference methods and non-interference methods. The angle is measured through the interference phenomenon caused by displacement or orientation changes in interference methods. The measurement accuracy is high, but the measurement range is generally small because the cross-sectional area in the angular direction of the measurement beam is small. Non-interference methods are based on the changes in the energy distribution of the light beam caused by orientation changes. The measurement accuracy depends on the resolution and stability of the detector. Therefore, the accuracy is generally lower but the measurement range is larger. Both solutions have their advantages and disadvantages depending on the application field.

#### 2.1.1. Non-Interference Methods

This chapter mainly introduces non-interference methods, which is commonly used when the accuracy requirement is not high, the structure needs to be simple, and the cost budget is low. From the perspective of classification, in the traditional laser field, the physical parameter changes directly related to the angle measurement value include the change in the spot position and the change in the total energy of the spot. The vision scheme is classified based on the angle measurement principle. Two typical schemes are introduced. One is to analyze the shape of the light spot to obtain the angle, and the other is to analyze the change of the color of the figure in the visual field to obtain the angle. In the field of optical frequency combs, the second-harmonic generation (SHG) method belongs to optical nonlinear effects and is the most expensive method in the non-interference category. In the quantum field, weak measurement is to obtain system information in a quantum state without significantly disturbing the quantum system. The obtained measurement results are informationally related to the disturbance, thereby obtaining angle information [24]. This method is more complex, but the achievable accuracy is very high. By designing corresponding absolute reference physical quantities, all of the above methods can achieve absolute measurement.

The optical dividing head [25] and angular polygons using multiple facets [26] are two types of solutions that were developed earlier, and there is currently limited research on them because of their low accuracy. The mainstream solutions primarily utilize a disc as the sensor base, which allows for the use of linear measurement schemes as angle measurement solutions. These solutions can be mainly categorized into two types: energy-based and phase-based.

**Figure 2:**
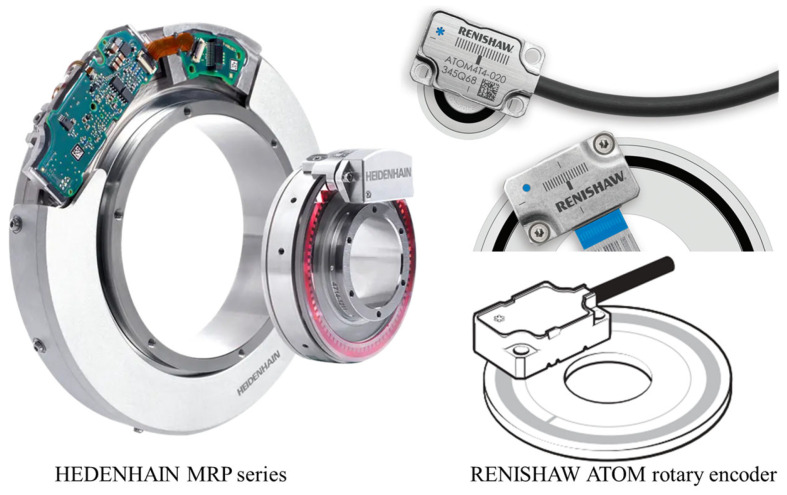
Two angle encoder products: accuracy@360°: up to ± 0.4″ (Heidenhain, Free State of Bavaria, Germany); up to ±1″ (Renishaw, London, UK).

Optical methods predominantly depend on the disk encoder scheme, commonly denoting the grating encoder arrangement, which employs a grating disk configuration for measurement purposes [27,28,29,30,31,32,33,34,35]. Previous researchers have conducted a lot of research on it, including structural optimization [36] and algorithm supplementary modifications [37,38,39,40,41,42,43,44,45,46]. The above method mainly relies on the scale of the code disk to measure large angles. The most important products on the market currently come from Heidenhain and Renishaw. There have been many additions to this type of product. Two angle encoder products based on holographic scales are shown in Figure 2. Detailed information can be checked on their official website.

On the one hand, large angle measurement generally involves the energy method, which changes the photoelectric energy at different angles. However, this simple energy utilization method has low accuracy [47,48,49,50,51] and relies on the stability and detection accuracy of the photoelectric itself [52,53]. On the other hand, phase-based methods allow for higher accuracy and better stability of measurements, e.g., calculating the angle change by monitoring the geometric length change of the light propagation [54] and laser self-mixing method [55,56]. The laser self-mixing method attains a resolution of 0.008 degrees within the measurement range of ±22 degrees [57]. Due to the fact that the measurement accuracy of phase changes can be achieved with a precision of 1/1000 cycle through mature interpolation methods, the angle accuracy of interferometric measurement and the Moire fringe method [49,58,59,60,61] are greatly improved compared to traditional optical methods [62].

**Figure 3:**
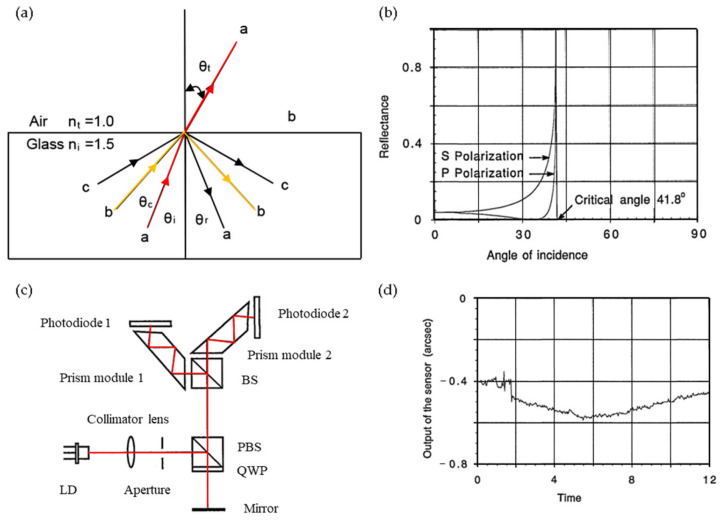
Angle measurement using internal reflection method: (**a**) internal reflection of light beams at an air–glass interface; (**b**) reflectance of internal reflection at an air–glass interface; angle of incidence is in degrees; (**c**) optical schematic of a prototype sensor [63]; (**d**) noise and drift of the prototype sensor. Time is in minutes. n_i_: higher-index medium (glass); n_t:_ lower-index medium (air); θ_c_ = arcsin (n_t_/n_i_); θ_i_: the angle of incidence.

The optical internal reflection method primarily relies on the variation in reflectivity at different incident angles. It exploits the internal reflection characteristics of a laser beam near the critical angle of an air–glass boundary. The fundamental approach involves employing a differential detection scheme to effectively minimize inherent nonlinearity in a reflectance–angle relationship. This enables the precise measurement of laser beam angular displacement through reflectance detection. A prototype sensor, demonstrating the capability to measure small angular displacements up to 3 arcmin with a resolution of 0.02 arcsec, possesses compact physical dimensions of only 50 × 50 × 25 mm and weighs a mere 70 g [63], which is shown in Figure 3. An additional significant advantage of this method is its versatility in sensor design for diverse applications. By adjusting key sensor parameters such as the initial angle of incidence, polarization state, and number of laser beam reflections, sensors can be tailored for wide-ranging or exceptionally high-resolution measurements.

**(A)**  **The location of the light spot**

**Figure 4:**
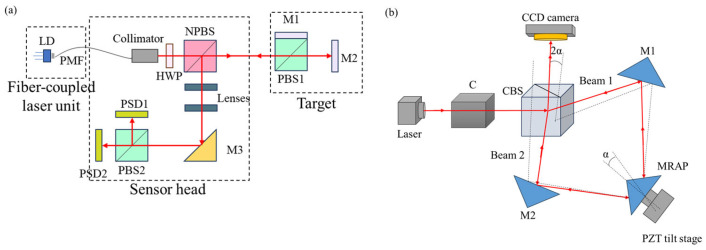
Autocollimator with expandability: (**a**) a laser-fiber autocollimation method can greatly reduce the laser beam drift [64]; (**b**) sensitivity improvement method [65]. PMF: polarization-maintaining fiber. LD: laser diode; HWP: half-wave plate; NPBS: nonpolarizing beam splitter; PBS: polarizing beam splitter; M: mirror; PSD: position detector; C, collimator; CBS, cube beam splitter; α, tilt angle given to MRAP (mirrored right-angled prism).

The autocollimator method is also used in single-axis measurement, as shown in Figure 4. This [64] method is different from the traditional mirror target but uses optical fibers and prisms to achieve angle measurement. Laser fibers are convenient pieces of equipment that can be used for measuring small angles with the difference in common-path polarized light [64] with the potential to be compact, and this method greatly reduces the laser beam drift caused by various factors and significantly improves the measurement accuracy and stability. In order to improve sensitivity, a Michelson interferometer has been developed, which uses a right-angled prism mirrored on the sides containing the right angle, to measure small tilt angles with double sensitivity compared to the classical Michelson interferometer. With this setup, it could measure tilt angles up to 1 μrad [65].

**(B)**  **The energy of the light spot**

**Figure 5:**
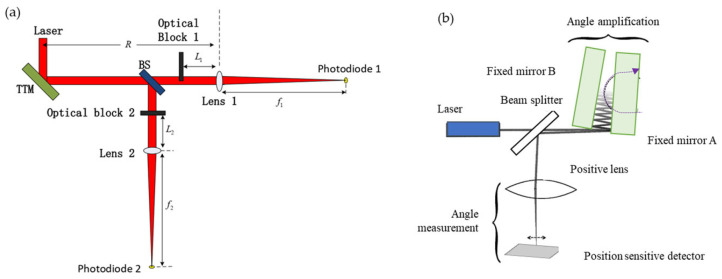
(**a**) Optical blocks in both directions to achieve angle measurement [66]; (**b**) angle amplification for nanoradian measurements [67].

The above methods are all used to detect changes in the position of the light spot. The energy detector methods are shown in Figure 5. Q Kong proposed an energy method that utilizes optical blocks in both directions to achieve angle measurement [66]. Another method is to use an angle amplifier to measure two fixed mirrors at a small angle [67].

**(C)**  **The visual method**

**Figure 6:**
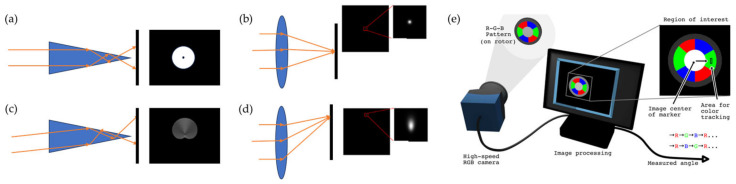
(**a**) Parallel light incident vertical to the axicon; (**b**) parallel light incident vertical to the convergent lens; (**c**) parallel light incident to the axicon at 8°; (**d**) parallel light incident to the convergent lens at 8° [73]; (**e**) concept of visual encoder [75].

The visual method mainly relies on the difference information of the image before and after the angle change shown in Figure 6, which can obtain the measurement results of the angle [68,69,70]. In addition, with the help of a special lens, different images that vary in angle can also appear [71,72]. Further, through different manifestations of light spots and machine learning methods, the relationship between angle and light spots can be established.

Liu et al. proposed a method in that an axicon forms a complex light spot with a clear edge, as well as rich feature information after total internal reflection and refraction on the sloped face compared with convergent lenses, which can only form a blurry edge spot [73]. Another method is to use a circular beam, which can simultaneously measure angle and distance [74].

Another solution is to directly design the detection target and associate the angle with the visual information on the target surface, for example, a measurement target with a disk mounted on a rotating disk. The measurement target uses color and pattern design to achieve absolute angle measurement [75,76]. The visual methods are shown in Figure 6.

At the same time, Li proposed an angle measurement method with the help of an infrared camera and a laser displacement sensor. At a distance of about 300 mm from the origin and within the field of view from −25° to 25°, the indirect measurement error of attitude (direction angle, pitching angle, and rolling angle) is 1.1°, which is a rough measurement within a larger angle range [77].

Vision solutions are mainly divided into point features, line features, regional features, multi-feature fusion, and other methods, as well as their corresponding artificial intelligence (AI) assistance methods [78].

The classic point feature extraction method extracts point features by identifying significant changes in color or grayscale in the image [79]. The line feature extraction method [80] involves the Line Segment Detector (LSD) straight line detection algorithm [81]. Compared with features such as points and lines, the area-based pose visual measurement method can make full use of more features on the target surface and is more robust to target pose measurements that are occluded or have symmetry [82]. Another example is traditional pose vision with multi-feature fusion [83,84].

In general, these visual image solutions can achieve multi-dimensional angle measurement, but their accuracy is generally on sub-degree scales, and the accuracy is low. The application fields are generally scenes where industrial accuracy requirements are not high.

**(D)**  **SHG method**

An optical frequency comb is a powerful optical tool that consists of a series of evenly distributed light pulses with very precise and regular frequency intervals [85]. This frequency interval is usually fixed and can correspond exactly to microwave or radio frequencies. The optical frequency comb interacts with the sample: the beam in the optical frequency comb is irradiated onto the sample surface, and part of the beam passes through nonlinear materials (such as second-harmonic crystals), doubling the frequency through the second harmonic. A spectrometer or other frequency domain analysis equipment can be used to perform spectrum analysis on the generated second-harmonic signal. Since the optical frequency comb provides a uniformly distributed frequency beam, the angular information of the sample surface can be obtained by observing the frequency component of the second-harmonic signal.

**Figure 7:**
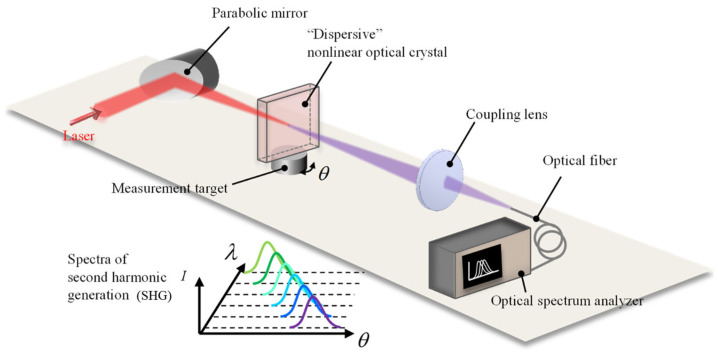
An optical frequency domain angle measurement system based on second-harmonic generation [87].

Based on the principle of angle dependence of second-harmonic generation (SHG) in nonlinear optics [86], an optical frequency domain angle measurement system based on second-harmonic generation is designed. The system structure is shown in Figure 7.

In this system, the laser generated by the mode-locked laser propagates as a common beam (FW), and the refractive index is expressed as $n_{0}$. The second-harmonic wave (SHW) is propagated as an extraordinary beam, and the refractive index is expressed as $n_{e}$. In order to avoid the chromatic aberration of the lens, the parabolic mirror is used to focus the laser beam on the nonlinear optical crystal and convert it into an SHW, which is focused on the multimode fiber through the lens, and the spectrum is recorded by the optical spectrum analyzer. According to the obtained spectral information, the angular displacement of the target can be calculated.

The intensity of the second harmonic can be expressed as follows [87]:

(1) $$P_{2}=\frac{8\pi^{2}d_{eff}^{2}}{n_{o}{\left(\lambda_{1}\right)}^{2}n_{e}\left(\theta ,\lambda_{2}\right)\varepsilon_{0}c\lambda_{1}^{2}}\frac{L^{2}}{S}P_{1}^{2}\sin^{2}\frac{\left|\Delta k\left(\theta \right)\right|L}{2}$$

where the effective nonlinear coefficient is denoted as $d_{eff}$, while $\lambda_{1}$ and $\lambda_{2}$ are wavelengths of the FW and SHW, respectively, $\varepsilon_{0}$ is the vacuum permittivity, c is the speed of light in a vacuum, S is the cross-sectional area of the focused beam, and L is the crystal length.

**Figure 8:**
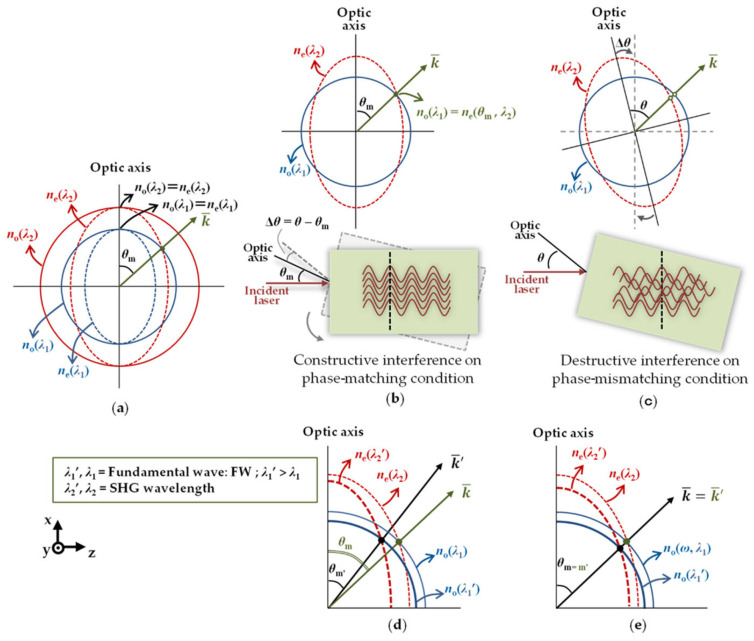
(**a**) Two-dimensional perspective of the refractive indices surface for type I negative uniaxial crystal; (**b**) phase-matching condition; (**c**) phase-mismatching condition; (**d**) the change in refractive indices’ surface followed by the change of phase match; (**e**) the change in refractive indices’ surface with the same phase-matching angle in a certain range of wavelengths [87].

The relationship between $n_{0}$ and $n_{e}$ is shown in Figure 8. When $n_{0}(\lambda_{1})=n_{e}(\theta_{m},\lambda_{2})$, the phase matching is satisfied. At this time, $\theta_{m}$ is the target deflection angle. The target deflection angle can be expressed as [87]

(2) $$\theta_{m}=\sin^{-1}\left(\sqrt{\frac{n_{o}^{-2}\left(\lambda_{1}\right)-n_{o}^{-2}\left(\lambda_{2}\right)}{N_{e}^{-2}\left(\lambda_{2}\right)-n_{o}^{-2}\left(\lambda_{2}\right)}}\right)$$

According to the above Equations, the intensity of the second-harmonic light (P_2_) decreases rapidly with the small angular displacement of the nonlinear crystal from the matching angle; this characteristic of the SHG can be employed for angle measurement.

**(E)**  **Weak measurement**

The basic measurement scheme has been introduced, but there are also other angle measurement schemes. The first one is quantum weak values. The concept of weak measurement was first proposed by Aharonov, Albert, and Vaidman in 1988. Unlike traditional quantum measurement methods, weak measurement can obtain measurement results that far exceed the eigenvalue spectrum of observable measurements [88].

**Figure 9:**
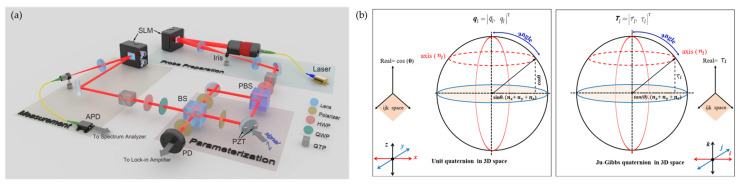
(**a**) Experimental setup for beam deflection measurement based on weak power recovery values [93]; (**b**) quaternion [96].

Quantum weak measurement makes two weak measurements of the system and uses the correlation between the measurements to obtain angle information. The sensitivity and accuracy of weak measurements can be enhanced through the appropriate design of pre- and post-operations. Quantum weak measurement has the advantages of high sensitivity and non-destructiveness, but its disadvantages, such as difficult experimental technology, limited information acquisition, and high analysis complexity, also need to be overcome. Therefore, it is necessary to weigh its advantages, disadvantages, and applicability in specific applications, and select appropriate measurement solutions and methods [89,90,91,92,93]. Reference [94] analyzes the basic principles of quantum weak value measurement technology, introduces the application research and progress in micro angle attitude measurement, and summarizes and analyzes the application trend of weak value measurement. Figure 9 shows the experimental setup, whose measurement accuracy is up to 4.08 nrad [93].

**(F)**  **Quaternion**

Another solution, although it also describes posture, uses quaternions as its description method. It is not surprising that this method can also express the state of the angle. Measurement methods for quaternions: a smaller number of mathematic operations performed in using quaternions for calculating angles, except for increasing performance, enables a decrease in rounding errors in calculation results that are accumulated in multiple measurements and may reach great values. Thus, the accuracy and performance of measurements increase with this method [95]. The experimental setup and quaternion are shown in Figure 9.

#### 2.1.2. Interference Methods

The interference method takes advantage of the wave nature of light and uses the intensity change of the light. After the reference beam and the measurement beam are combined to obtain the change in phase with the change in displacement, the displacement is converted into angle changes. According to different light sources, it can be divided into three methods: homodyne, heterodyne, and optical frequency combs. Among them, homodyne interference only requires one laser light source with stable frequency, while heterodyne interference requires two light sources with different frequencies. In comparison, the cost of homodyne angle measurement is moderate, but the heterodyne method is more accurate. In these two methods, absolute measurement is difficult because the signal changes periodically and an absolute zero position cannot be added. However, the optical frequency comb method can more conveniently achieve absolute angle measurement.

**(A)**  **Homodyne interference**

**Figure 10:**
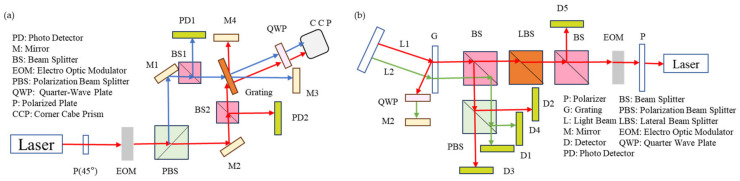
(**a**) Coplanar grating interferometer [116]; (**b**) double-diffraction grating interferometer [115].

Laser interferometry is a measurement method based on the principle that the laser phase changes when the angle changes. This method of measuring the phase is generally more accurate, but the corresponding set of equipment is also more expensive and complex. This method generally has a small range but high accuracy [6,9,97,98,99,100,101,102,103,104,105,106,107,108,109,110,111,112], and in order to further investigate its mechanism and error correction, an independently characterized angle measurement system that is useful for angle calibrations is proposed [113]. As for the environmental effect, measurement sensitivity can be improved through immersing the beta-barium borate plate in grapeseed oil, which can avoid deliquescence in humidity [114]. The heterodyne interference method in a coplanar plane can achieve a high resolution [115], as shown in Figure 10. Experiment results show that the angle resolutions of the system were found to be 33.7 nrad, while the values of repeatability were found to be 33.7 nrad [116]. The homodyne interference methods are shown in Figure 10.

**(B)**  **Heterodyne interference**

**Figure 11:**
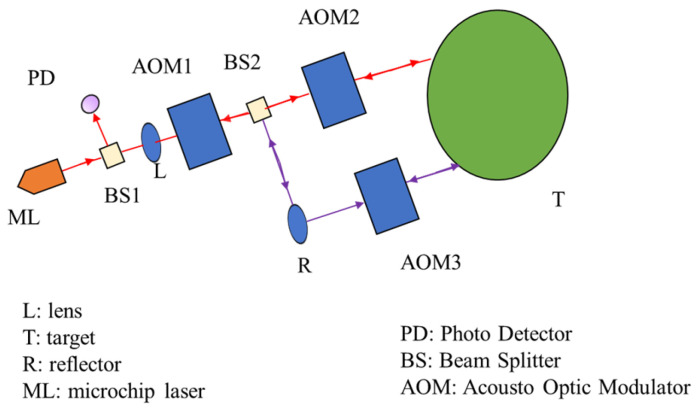
Diagram of the optical method for linear and angular measurement [118].

Compared with homodyne interference, heterodyne interference has higher stability and accuracy [10,117], as shown in Figure 11. Xin Xu proposed a dual-beam heterodyne method based on feedback interferometry for the non-contact measurement of linear and angular displacement, and the experimental results show that the prototype has a stability of 0.15″ over 1 h with a resolution of 0.02″. The linearity is 1.34 × 10^−4^ in the range of 360° [118]. The homodyne interference methods are shown in Figure 11.

**Figure 12:**
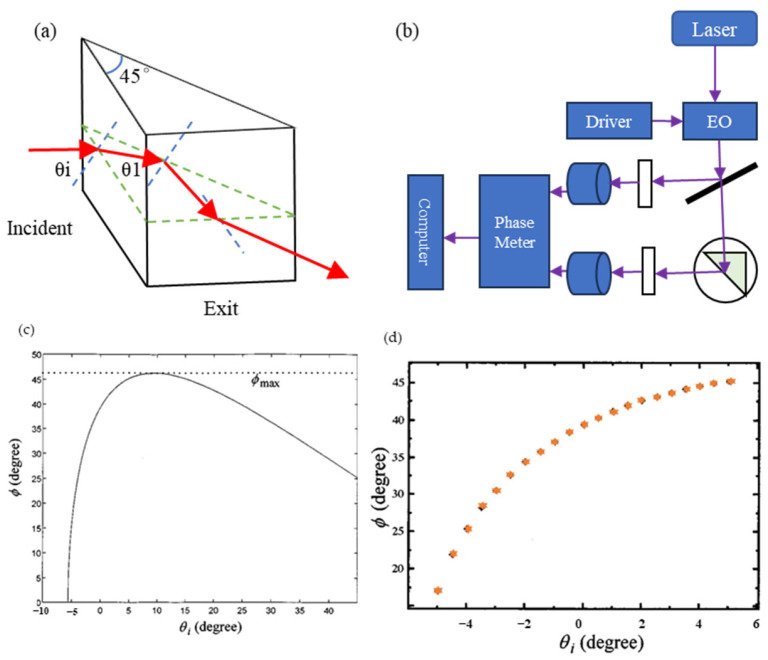
Angle sensors based on the relationship between the phase difference and the incident angle at total internal reflection: (**a**) the total internal reflection in a right-angle prism; (**b**) schematic diagram for this novel method for measuring small angles based on TIRHI. EO: electro-optic modulator; D: photodetector; AN: analyzer. (**c**) The curve of Φ (phase difference) versus θi (incident angle); (**d**) the experimental curves of Φ versus θi [6].

There are many interference methods [119] that are variations of the non-interference method. In contrast to Huang’s method [63], a novel optical method for angle measurement based on total internal reflection heterodyne interferometry (TIRHI) was proposed [6] and is shown in Figure 12. In TIRHI, the phase difference between the s and p polarization states during total internal reflection is measured via heterodyne interferometry. The small-angle measurement is achieved by evaluating only the phase difference, which varies depending on the incident angle. This method offers several advantages over other methods. Firstly, the phase difference is independent of intensity and can be accurately extracted despite surrounding light and light source instability. Secondly, the common-path configuration provides high stability against air turbulence. Thirdly, the optical path setup is simple, compact, and easy to align. Additionally, the method provides a high resolution (8 × 10^−5^ deg) and a larger measurement range (10 deg).

In traditional interferometer solutions, in order to be able to measure angles, the displacement is usually first calculated using the phase, and then the angle is solved [120].

**Figure 13:**
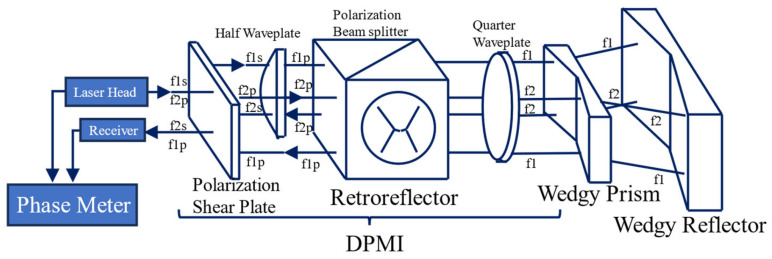
Schematic of roll-angle measurement system [120]; f: different frequency; DPMI: differential plane mirror interferometer.

A precision roll angle measurement system based on the differential plane mirror interferometer (DPMI) is proposed and shown in Figure 13. This system utilizes a DPMI with a wedgy angle prism and a wedgy angle reflector to generate diagonal symmetry of the two frequency beams. The residual of the nanopositioning stage and roll angle system is found to be less than 1 μrad, thus confirming the accuracy of the measurement principle. Consequently, the stability of the roll angle measurement system is deemed satisfactory, with an average deviation of measurement experiments being less than 5 μrad.

**(C)**  **Optical Frequency Comb method**

**Figure 14:**
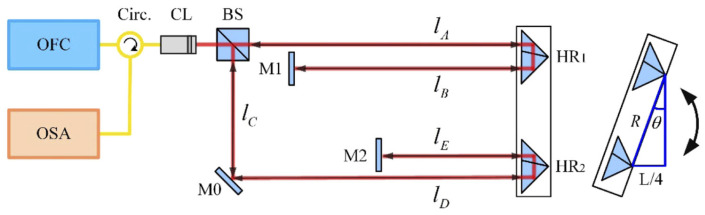
Schematic diagram of the absolute angular measurement [121]. OFC, optical frequency comb; Circ., fiber circulator; CL, collimator; BS, beam splitter (50:50); M0, M1, M2, plane mirrors; HR1, HR2, hollow retroreflectors; OSA, optical spectrum analyzer. The red line corresponds to the light that propagates in the free space and the yellow line corresponds to the light that propagates in the single-mode fiber.

In the method of measuring the absolute angle of dispersion interference using an optical frequency comb, two measuring arms are constructed by a parallel configuration composed of a plane mirror and an inverse mirror. It can make full use of the non-fuzzy metric of the dispersion interferometry to achieve absolute angle measurement in a wide range [7,8,9]. The system structure is shown in Figure 14.

An optical frequency comb (OFC) is a kind of ultra-short pulse laser with stable period output by mode-locked laser. This pulse function can be expressed by the Fourier series. In the frequency domain, it is a series of longitudinal mode sequences with equal intervals. Each longitudinal mode corresponds to one term of the Fourier series [122]. Optical frequency combs have developed rapidly to support the most accurate atomic clocks in the world [123,124]. Nowadays, optical frequency combs have developed into one of the most active fields in photonics [125,126], which has a significant impact on the development of science and technology [127,128,129].

When the pulse sequence of an OFC irradiates a typical Michelson interferometer, the interference spectrum is sampled and analyzed by an optical spectrum analyzer (OSA). In order to avoid the influence of dispersion on absolute angle measurement, a hollow-corner cube prism is used instead of a solid-corner cube prism as the target mirror. When the target mirror rotates, the two light beams reflected by the hollow retroreflector (HR) and the reflector recombine and interfere with each other, and the interference signal is sampled and analyzed by the OSA. The rotation angle of the target mirror can be expressed as [121]

(3) $$\theta =\arcsin\left(\frac{L}{4n_{g}R}\right)$$

where R is the length of the sinusoidal arm, which represents the fixed spacing between the two resonance peaks. L represents the optical path difference between the two interference arms. It is worth noting that $n_{g}=n+(dn\;/\;dv)\;v$ represents the group refractive index of air, which is determined by the center frequency of the light source, and n is the refractive index of air.

**Figure 15:**
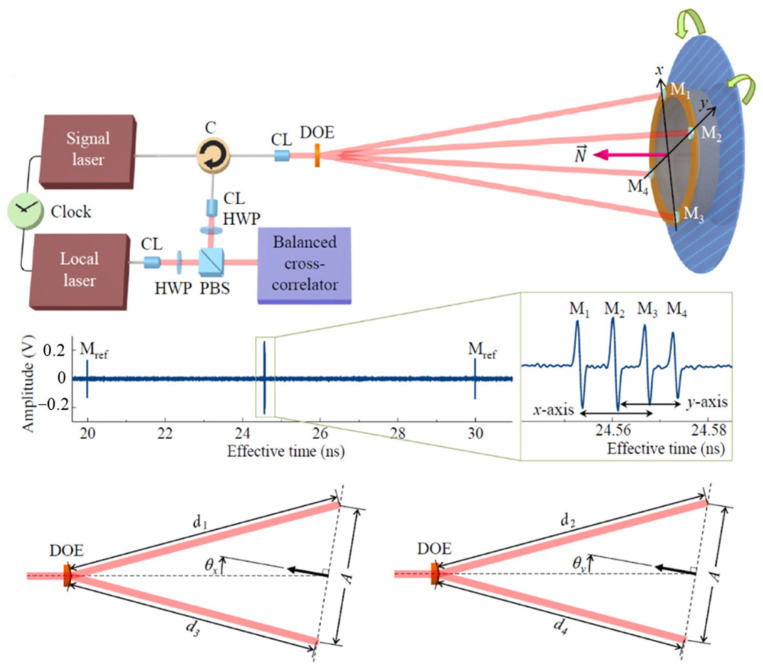
An example of the angle measurement based on the absolute distance measurement [130]. C: circulator; CL: collimator; DOE: diffractive optical element; HWP: half-wave plate; PBS: polarizing beam splitter; M: target mirror.

An extension scheme of an absolute distance interferometer using femtosecond laser pulses for multi-degree-of-freedom sensing is proposed in Figure 15 [130]. The measurement system is based on a dual-comb interferometer and uses two femtosecond lasers, which are called signal lasers and local oscillator lasers, respectively. Signal light is the main light source for time-of-flight measurement, and frequency down-conversion sampling requires local oscillator light with different repetition frequencies. The measurement system can measure the angle while measuring the absolute distance [131]. The angle is expressed as follows [130]:

(4) $$\left\{\begin{array}{c} \theta_{x}=sin^{-1}\left(\frac{d_{1}-d_{3}}{A}\right)\\ \theta_{y}=sin^{-1}\left(\frac{d_{2}-d_{4}}{A}\right) \end{array}\right.$$

where A is the distance between the corresponding two mirror reflectors; $d_{1},d_{2}{,d}_{3},d_{4}$ is the distance from the diffractive optical element to the target mirror.

### 2.2. Mechanical Methods

Mechanical methods mainly use angle measurement with the help of mechanical structures. Representatives of this measurement scheme mainly include the cooperation of gear pairs and the performance changes of mechanical structures with angle changes. Compared with optical and other methods, these methods are not very accurate, and the measurement range can be large or small, but the advantage is that the scheme is simple, cost-effective, compact, and robust.

Cao, K. proposed a novel measurement method that applies the clock pulse counting technique to assess the transmission error of the gear pair in an input-and-output angle encoder, while simultaneously accounting for the inherent transmission error of the gear pair [132], and the measurement precision reached 1 arcsecond in the tooth frequency. Li, Y. combined multi-toothed discs with the method of dual-frequency laser interference [133], and the measured error can be less than 0.5″ in every ±5° range.

**Figure 16:**
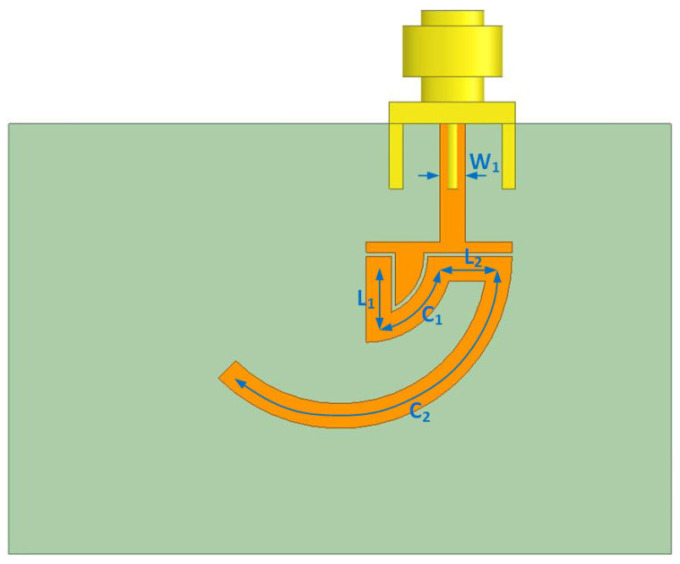
The layout of the utilized half-wavelength resonator; C_1_ is a 90° circular sector line and C_2_ is a θ° circular sector line [134].

Other methods mainly include angle measurement solutions that rely on special materials or structural properties. A new inclinometer concept based on the angular half-wavelength microstrip resonator is shown in Figure 16. The inclinometer is composed of a microstrip transmission line resonator on a printed circuit board and a second transmission line on an overlapping circuit board that rotates according to the inclination angle. The resolution shown in the experiments is 0.035° [134].

### 2.3. Electromagnetic and Inertial Methods

**(A)**  **Electromagnetic method: inductance, capacitance, and Hall effect**

**Figure 17:**
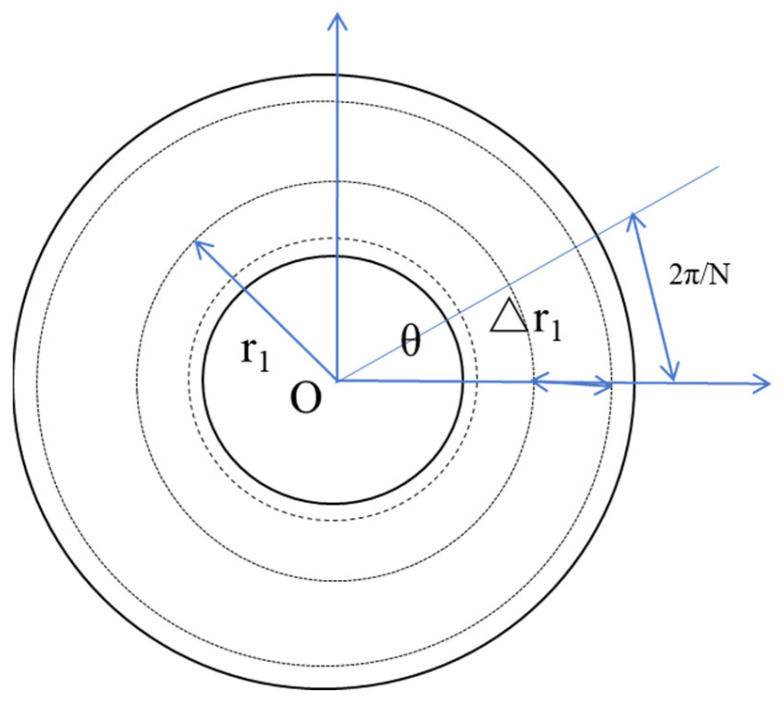
Schematic diagram of a single-stage capacitive angular displacement sensor [31].

The inductance method uses the electromagnetic induction that occurs during displacement for measurement. Wu, L. proposed an inductive sensor for angle measurements of large and hollow rotary machinery. The experiment results showed that the measurement accuracy of the sensor prototype is ±40″ during the measurement range of 0°–360° [135]. In research endeavors, electromagnets are commonly employed to accomplish the encoding function and can facilitate absolute measurement [136]. This electromagnetic type can achieve an angle measurement similar to that of a photoelectric disc encoder [137,138,139,140,141]. Time grating uses a capacitive angle encoder, shown in Figure 17, and the final accuracy is within ±4.4″ over a full 360° range, which can also be used for displacement measurement [28,29,30,31,142,143,144]. There are also sensors with the Hall effect [145], and the angle measurement results show an average measured error of ±1.263°. Specific content on the Hall effect can be seen in reference [1].

**(B)**  **Inertial method**

The principle of inertial sensors is based on the inertial characteristics of objects, mainly including gyroscopes and accelerometers. Gyroscopes use the law of conservation of angular momentum to measure the angular velocity of an object. Generally, a gyroscope consists of a rotating inertial element and a sensing device. When an object rotates, the inertial element will be subject to rotational torque, and its rotational angular velocity relative to the reference coordinate system will cause changes in the signal output by the sensing device. By measuring these signal changes, the angular velocity and angle of the object can be inferred.

Accelerometers indirectly infer angular changes by measuring the acceleration of an object. Accelerometers typically use microelectromechanical systems (MEMS) technology, which contain tiny systems of masses and springs. When the object’s acceleration changes, the mass will be displaced by a force, resulting in the deformation of the spring. By measuring the deformation of the spring, the acceleration of the object can be obtained. By integrating the acceleration, the displacement can be obtained and the angle can be further inferred [146,147,148,149,150,151,152,153].

These inertial sensors can be used alone or integrated into an inertial navigation system (INS). INSs can achieve more accurate angle measurement and positioning by fusing data from multiple inertial sensors, including gyroscopes and accelerometers. However, inertial sensors also have some limitations, such as drift, error accumulation, and other problems, so calibration and error correction are required in specific applications.

**Figure 18:**
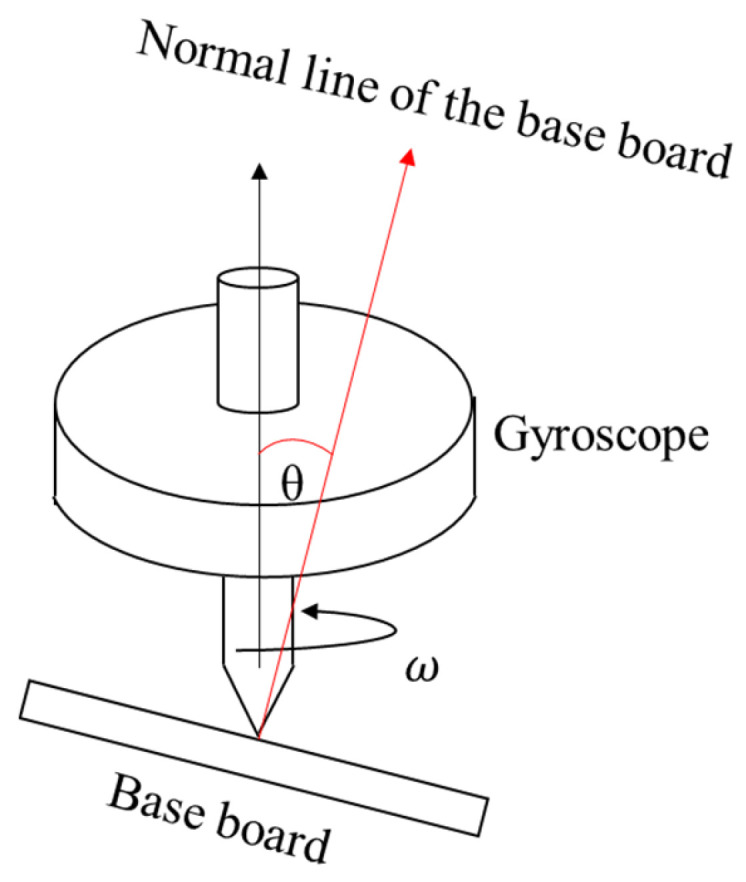
Principle of the gyroscope structure.

When the gyroscope is in free motion, its rotation axis will remain unchanged, as shown in Figure 18. Based on this principle, the gyroscope measures the angle of an object in space.

The Laser Gyro is a device that uses the principle of laser interference to measure the angular velocity of rotation. It consists of a ring laser in which the laser beam propagates in two opposite directions in a ring optical path and interferes at the intersection of the optical paths. When the gyroscope rotates, the beam propagation time will vary slightly due to the rotation as a result of the Sagnac effect. By measuring this time difference, the speed and direction of the object’s rotation can be determined [154,155]. Fiber optic gyros use the Sagnac effect when light propagates in optical fibers to measure rotation angular velocity. It consists of a fiber optic ring light path and a laser, in which interference occurs when light propagates in clockwise and counterclockwise directions. By measuring the phase difference between interference patterns in two directions, the rotation speed and direction of an object can be determined. Fiber optic gyroscopes have the advantages of high precision and resistance to vibration and are widely used in navigation systems, aircraft control, inertial navigation, and other fields [156,157]. The biggest difference between the two is whether there is an optical fiber as the propagation path of the light beam. Since the existence of optical fibers can isolate external interference, they have higher stability, precision, and resolution, and the overall volume and weight are reduced.

In order to eliminate constant drift error and improve the angular rate measurement accuracy of magnetically suspended control and sensing gyroscopes (MSCSGs), a high-precision angular rate measurement method based on component-level rotation modulation is proposed in this article [158].

The measurement method of using image analysis of angles has been introduced by predecessors, and even deep learning methods [78] directly read the angle changes of the measurement target and then perform image analysis to obtain angle results [76,159,160,161,162,163,164,165]. By utilizing the characteristics of optical fibers, the measurement of large-angle autocollimators can be achieved [166], with a measurement range of −100 to 100 arcmin [167] and reaching 110 degrees [168].

**Figure 19:**
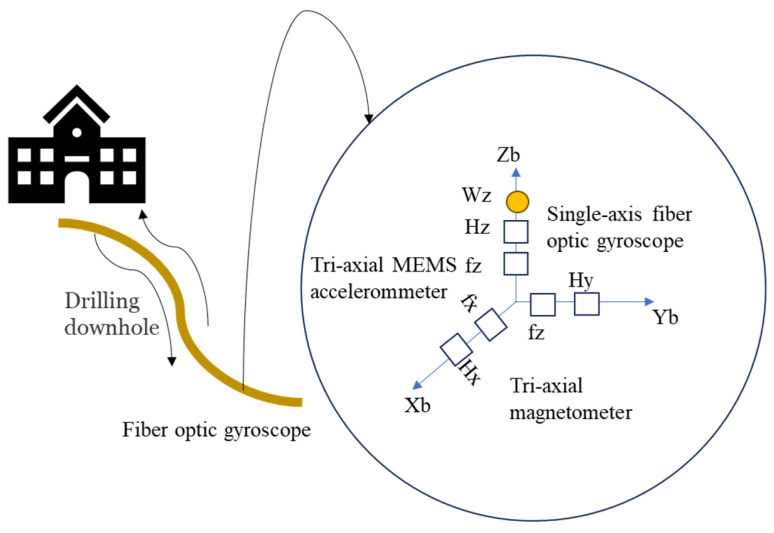
Schematic of the novel attitude-measurement-while-drilling system [169].

A novel attitude-measurement-while-drilling system is designed, which consists of a single-axis fiber optic gyroscope (FOG), a tri-axial micro-electromechanical system (MEMS) accelerometer, and a tri-axial magnetometer. The schematic of the novel attitude-measurement-while-drilling system is shown in Figure 19, of which the theoretical error is less than 0.38°. Three real-time estimation experiments of the magnetic heading angle were carried out; the overall estimation error did not exceed 0.35°, and the average estimation error did not exceed 0.3° [169].

## 3. High Precision with Multiple Axes

High-precision multi-axis measurement methods can be classified according to different measurement targets. Generally, as a measurement target, a plane mirror can mainly achieve angle measurement with two degrees of freedom, but cannot achieve the measurement of roll angles. Therefore, subsequent researchers have proposed gratings as measurement targets. This target can measure three angles using the property of diffracted light that is sensitive to roll angles. Unfortunately, its nonlinear relationship when measuring at large angles will lead to angle measurement error. The high-precision measurement of multi-dimensional angles is the premise of error correction. The sharp decline in accuracy will affect the correction effect. Therefore, researchers have proposed many new self-designed targets, which can achieve multi-dimensional angle measurement while improving the measurement range and measurement sensitivity.

### 3.1. Planar Mirror Target

**Figure 20:**
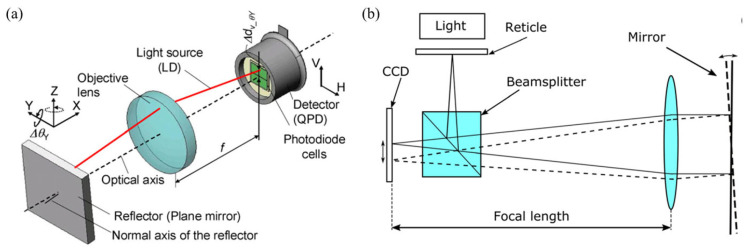
Mirror: (**a**) the non-interference method [170]; (**b**) the interference method [171].

An autocollimator is a device that measures the angle by measuring the lateral displacement of the light spot received by the detector during the angular displacement of the beam, which is shown in Figure 20. When an autocollimator is in operation, a collimated light beam is first projected onto a target, which reflects the collimated light back and projects it onto the detector of the autocollimator. If the surface being measured is perpendicular to the direction of incidence of the autocollimator’s beam or the angle being measured is perpendicular, the autocollimator light will return to the same position. However, if there is a tilt on the surface or the angle changes, the collimated light will deviate from its original position. This deviation will result in the observation of a light spot displacement on the detector and obtain the angle value.

Based on the mirror, the pitch and yaw angle can be measured. The range is ±1000″ for both axes. The estimated standard uncertainty for the full range is 0.0036″ for the horizontal direction and 0.0053″ for the vertical direction [171].

**Figure 21:**
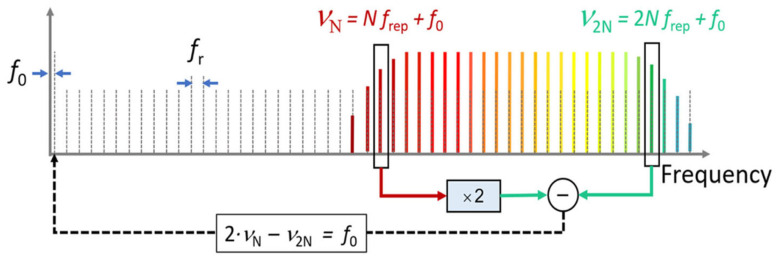
Offset frequency detection via self-referencing [126].

Optical frequency combs have a trend of application in the field of angle measurement due to their good time repeatability and phase stability; optical frequency combs can be generated by a mode-locked femtosecond laser. The frequency of the mode-locked laser has two adjustment parameters, which are the repetition frequency, $f_{rep},$ and the carrier phase offset frequency, $f_{0}$. The repetition frequency controls the ‘comb distance’ of the output optical frequency comb, and the carrier phase offset frequency controls the ‘comb tooth slip’. When both $f_{rep}$ and $f_{0}$ are fixed, the mode-locked laser outputs an optical frequency comb [172], as shown in Figure 21 [126].

At the same time, a femtosecond laser autocollimator is established in combination with laser autocollimation [173,174,175,176]. In addition, displacement measurement methods based on the optical frequency comb can also be used for angle measurement; for example, a 0.073 arcsec repeatability was obtained for angle measurement by measuring the parallelism of the absolute distance of multiple targets through the flight time [130].

**Figure 22:**
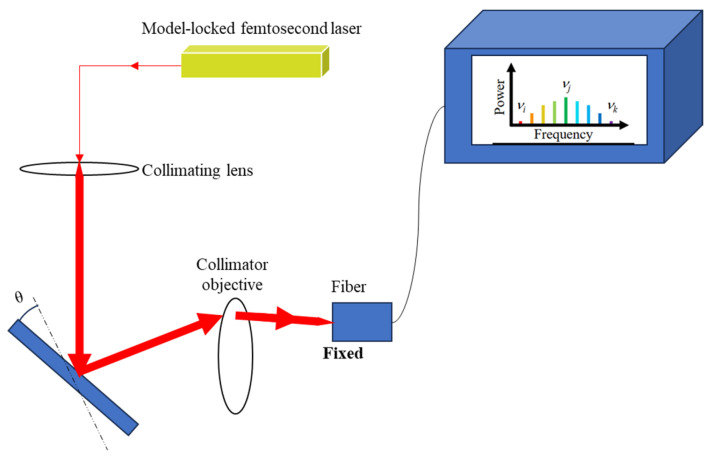
The optical device schematic diagram of the angular displacement measurement method based on the lens chromatic aberration phenomenon [177].

According to the principle that different frequencies of laser project onto a collimated lens with chromatic aberration to produce different refractions [177], some scholars have established the angle sensor, as shown in Figure 22. The sensor injects the mode-locked laser beam into the collimating objective lens with chromatic aberration. Each optical mode in the optical frequency comb incident on the lens refracts and propagates at different angles, and then uses the optical fiber with the spectrometer as the photodetector to collect the optical signal. The collected signal is analyzed by spectral analysis, and the optical mode *j* corresponding to the maximum spectrum before deflection is recorded. The optical mode *k* corresponding to the maximum spectrum after a small angle deflection of a reflector is recorded. According to the change in the focused spot before and after deflection, the calculation formula of the deflection angle can be obtained as follows [177]:

(5) $$∆\theta =\frac{1}{2}tan^{-1}\left(\frac{f_{j}}{f_{k}}tan2\theta_{0}\right)-\theta_{0}$$

where $\theta_{0},f_{j},\;and\;f_{k}$ are the design parameters of the optical system, the corresponding computable parameters under optical mode j, and the corresponding computable parameters under optical mode k, respectively.

### 3.2. Grating Target

**Figure 23:**
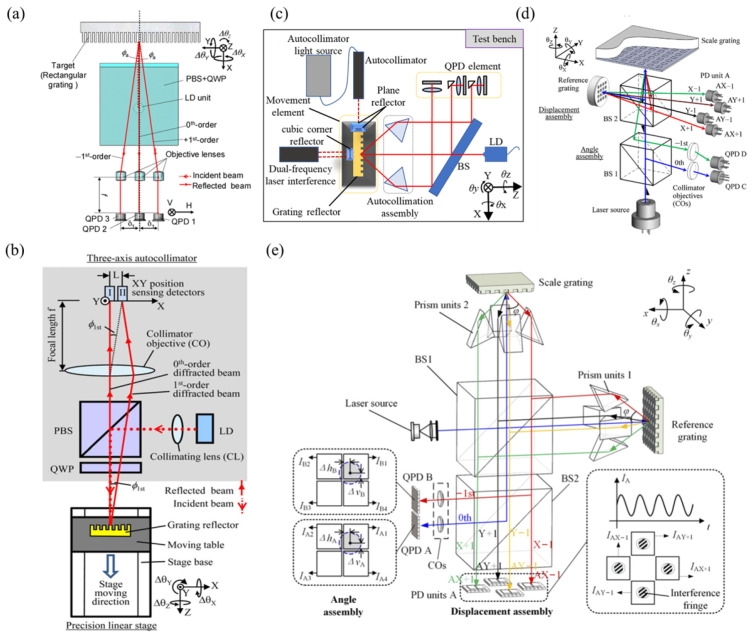
Grating: (**a**) three beams of diffracted light [170]; (**b**) two beams of diffracted light [178]; (**c**) four-DOF test bench based on the grating target [179]. (**d**) The six-DOF grating encoder [17]. (**e**) Operating principle of the single-channel six-DOF grating encoder [16]. LD: laser diode; QWP: quarter-wave plate; PBS: polarizing beam splitter; QPD: quadrant photodiode detector.

In the grating measurement scheme, the measurement mainly relies on the changes in the beam direction of the three diffracted lights as the position and orientation of the grating change. They are shown in Figure 23. The three-beam diffracted light scheme can compensate for each other with the help of positive and negative first-order diffracted lights, with high accuracy as shown in Figure 23a, while the two-beam diffracted light scheme uses fewer detectors and is less expensive, as shown in Figure 23b.

**Figure 24:**
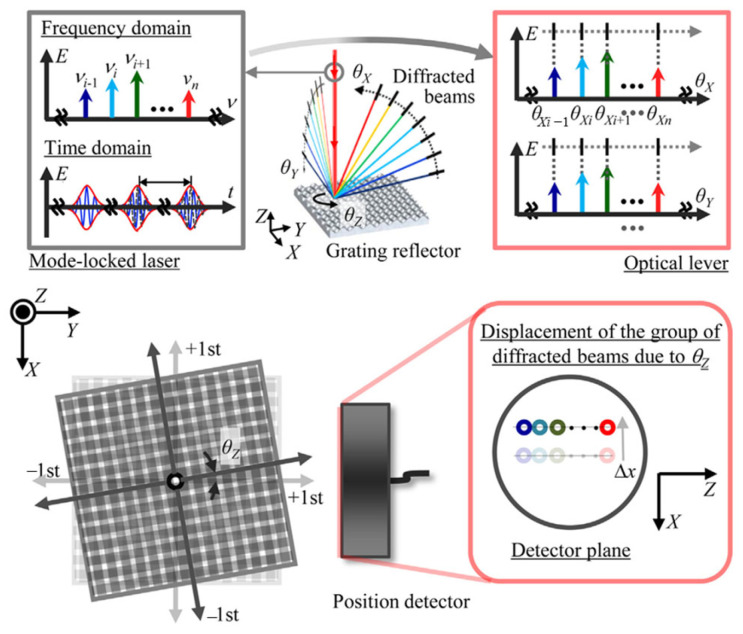
The schematic diagram of angular scale comb generated by a mode-locked femtosecond laser [199].

Gratings are currently used in various fields due to their excellent structural dimensional consistency and structural stability thanks to increasingly advanced processing methods [180,181,182,183,184,185,186,187,188,189,190,191,192,193] as a standard device widely used in precision displacement [194,195,196,197] and orientation measurement [178,179,198]. The optical frequency comb output by the mode-locked femtosecond laser can be used to generate an ‘angle standard comb’ as an optical scale for angle measurement. A new concept of absolute angle scale is proposed by enhancing the dispersion characteristics of diffraction grating [199]. The principle of the angular scale comb generated by the mode-locked femtosecond laser is shown in Figure 24. The enhancement of the dispersion characteristics of the diffraction grating [200] produces the angular scale comb [11].

In a mode-locked laser, each mode has a specific frequency, and each first-order diffraction beam of the diffraction grating has a diffraction angle corresponding to the frequency of each mode. The diffraction angle of the first-order diffracted beam of the ith optical mode in the mode-locked laser can be expressed as follows [199]:

(6) $$\theta_{i}=arcsin\left(\frac{c}{n_{air}gv_{i}}\right)\left(i=1,2,3,\ldots ,n\right)$$

where $c,n_{air},g{,\;and\;v}_{i}$ are the light velocity in vacuum, the refractive index in air, the grating period, and the frequency of the ith optical mode in the mode-locked laser, respectively.

**Figure 25:**
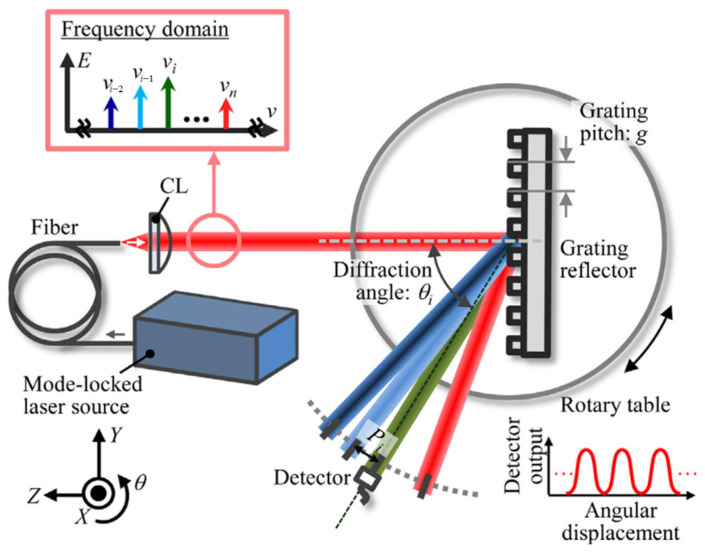
An example of the angle comb used for angle measurement [199].

The angular motion of the grating reflector around the X, Y, and Z axes can be measured by using the first-order diffraction beam as the scale of angle measurement. Figure 25 shows an example of the angle comb used for angle measurement. The reflective grating is installed on the turntable, and the detector detects the change in the angle comb with the rotation of the reflective grating to monitor the angular displacement of the turntable. However, there is a trade-off between the resolution of angle measurement and the size of the optical path. It is difficult to achieve high-resolution angle measurement in some scenarios requiring small-sized optical paths.

**Figure 26:**
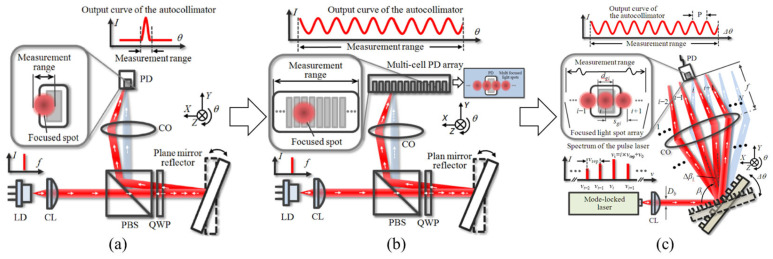
A comparison between the conventional laser autocollimator and the femtosecond laser autocollimator: (**a**) A laser autocollimator with a single-mode laser source employing single light position-sensing photodiode; (**b**) A laser autocollimator with a single-mode laser source employing multi-unit light position-sensing photodiodes;(**c**) A femtosecond laser autocollimator with a mode-locked femtosecond laser source [176]. CL: collimating lens; CO: collimator objective; PD: position-sensing photodiode.

The traditional angle measurement uses a laser diode as the light source and a plane mirror as the mirror. A single-light PSD with a high response speed is used to detect the spot position [178,201]. The structure is shown in Figure 26a. However, due to the limited size of the focused spot and the sensitive area of the PSD, the angle measurement range is limited to 100 arcseconds [202]. Some scholars have proposed a method using a multi-unit PSD array to expand the angle measurement range of traditional laser autocollimators. The structure is as shown in Figure 26b. *N* PD units (multi-cell PD array) can increase the angle measurement range to *N* times the original, and the measurement range can be expanded to 2300 arcseconds [203]. However, due to the complexity of the production process and the poor stability of the measurement system, it is hard to industrialize.

After the angle measurement method of using a mode-locked laser combined with reflective diffraction grating is proposed, some scholars have carried out further research and designed a complete angle measurement method [176], as shown in Figure 26c. Different from the single-wavelength LD, the spectrum of the mode-locked laser is composed of a series of optical modes arranged equidistantly in a wide frequency range. The mode-locked laser beam is collimated by a collimating lens (CL) and projected onto a tilted reflective diffraction grating. The relationship between the ith mode of the incident mode-locked laser and the diffraction angle of the ith first-order diffraction beam can be expressed as follows [176]:

(7) $$\beta_{i}=arcsin\left(\frac{c}{n_{air}gv_{i}}-sin\alpha \right),\left(i=1,2,3,\ldots ,N\right)$$

where $c,n_{air},g{,v}_{i},\;and\;\alpha$ are the light velocity in a vacuum, the refractive index in air, the grating period, the frequency of the ith optical mode in the mode-locked laser, and the angle of incidence of the collimated mode-locked laser beam with respect to the normal of the grating reflector, respectively.

**Figure 27:**
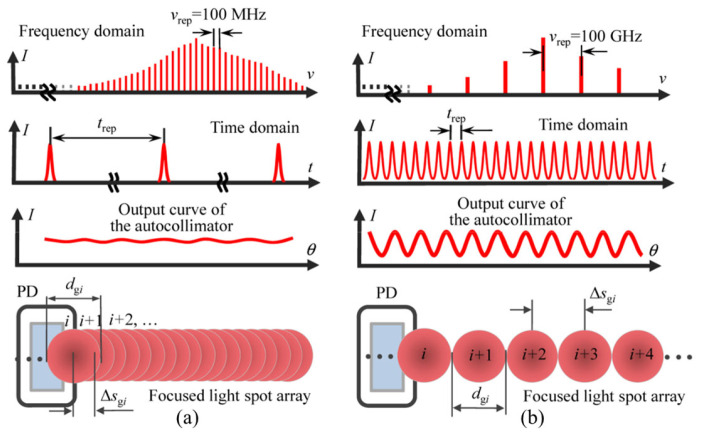
Schematic of characterization of the focused light spot array by a mode-locked laser: (**a**) low repetition rate; (**b**) high repetition rate [176]. PD: position-sensing photodiode.

The focal spot array detected by the PSD can expand the angle measurement range, and the maximum measurement range can reach 11,000 arcseconds, which is much larger than the measurement range of a traditional laser autocollimator. It should be noted that the repetition frequency of the mode-locked laser has a great influence on the focusing spot separation. The mode-locked laser with a high repetition frequency should be used [176]. When the repetition frequency is greater than 100 GHz, the spot overlap shown in Figure 27a can be avoided, and the separated spot shown in Figure 27b can be obtained.

The repetition rate of most commercial mode-locked femtosecond lasers is in the order of 100 MHz. When this laser source is used in the laser autocollimator, the distance between adjacent spots in the focal plane of the collimating objective is several nanometers, which cannot be distinguished separately. In most cases, adjacent spots will inevitably overlap with each other, resulting in inaccurate reading of the autocollimator.

**Figure 28:**
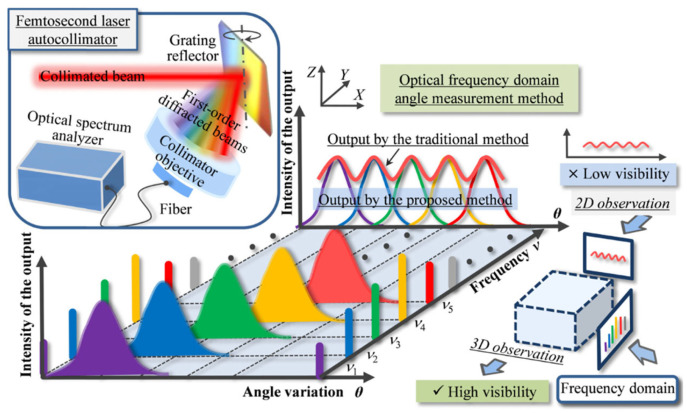
Principle of the optical frequency domain angle measurement method associated with the femtosecond laser autocollimator [173].

Using an optical spectrum analyzer (OSA) instead of a PSD to detect overlapping focused first-order diffracted light can solve the above problems [173,174]. As shown in Figure 28, the optical frequency determined by the focused first-order diffracted beam is the optical frequency of the transmitted light corresponding to the repetition frequency interval on the modulated femtosecond laser spectrum. Under the condition that the frequency resolution of the OSA is less than the mode interval of a femtosecond laser, the OSA can clearly distinguish the output curve of each order of the diffracted beam with respect to the angle change of the grating mirror in the frequency domain.

According to the simulation results, the visibility of the traditional method of using PSD to detect the focused first-order diffraction beam is only 5.1%, which is much lower than the 100% visibility detected by the spectrometer. The visibility is defined as [173]

(8) $$V_{visibility}=\frac{{I_{max}}_{i}-{I_{min}}_{i}}{{I_{max}}_{i}+{I_{min}}_{i}}$$

where ${I_{max}}_{i}$ and ${I_{min}}_{i}$ are the maximum and minimum output values of the autocollimator, respectively, corresponding to the ith optical mode of the femtosecond laser.

Table 1 shows the highest performance of the OFC method with the measurement range, resolution, and cost. The new angle measurement technology based on an optical frequency comb is expected to achieve the performance that traditional methods cannot achieve.

### 3.3. Self-Designed Target

The accuracy and resolution of the autocollimator detection primarily rely on the sensitivity of the sensor [205]. It is imperative to minimize the noise level of the sensor signal in order to achieve high-resolution angular displacement measurement [206,207,208]. The method of self-collimation has maintained enduring popularity and continues to be utilized, with the development of numerous diverse variations over time [171,173,178,209,210,211,212,213,214,215,216,217,218,219,220,221,222,223,224,225,226,227]. The detection units are CCDs (Charge Coupled Devices) [228,229], QPDs (quadrant photodiodes) [230,231], and PSDs (position-sensitive detectors) [64,232,233]. In multi-degree-of-freedom measurement, an autocollimator is often used for integrated design, with the help of PSDs [234,235,236] and QPDs [179,237]. The PSD also needs position correction at long distances and high-speed measurements [238,239].

**Figure 29:**
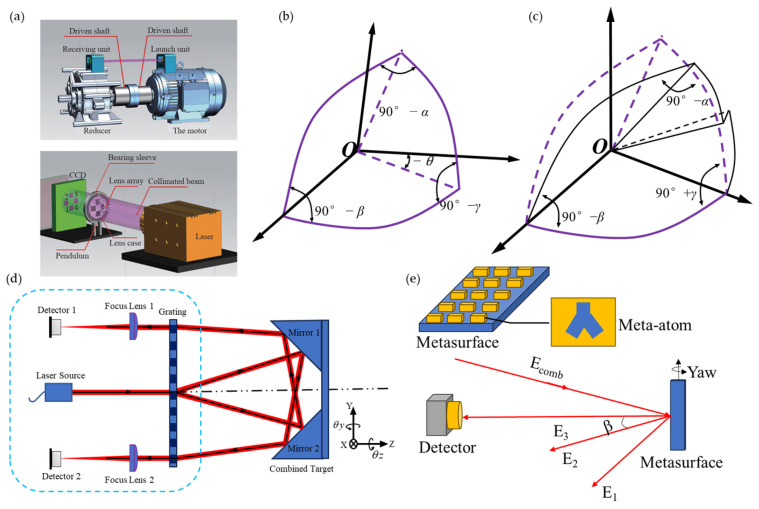
Different detection targets based on autocollimators: (**a**) self-designed target [240]; (**b**) hollow cube corner reflector (HCCR) [225]; (**c**) modified cube corner reflector (MCCR) [241]; (**d**) self-designed target [232]; (**e**) the metasurface target [242].

Based on the principle of autocollimators, various measurement methods have been developed for different detection targets, such as angle measurement schemes based on gratings [178], planar mirrors [171], and self-designed target surfaces [232], which are shown in Figure 29.

The design of a flat mirror as a detection target generally cannot achieve the measurement of roll angles but has an advantage of low cost [171]. The grating and self-designed target surface measurement methods based on the autocollimator can achieve angle measurements of three-dimensional attitude, mainly using the CCD method [240,243], and these methods generally require calibration [12,13]. Metasurface angle encoders are artificially designed materials with micro/nano-scale structures that can be used to control the propagation and reflection of light. Metasurface encoders utilize the special structure of metasurfaces to encode and decode optical signals and obtain angle information [242]. The metasurface method can also measure displacement [244,245,246], which is able to obtain angle information in the future.

**Figure 30:**
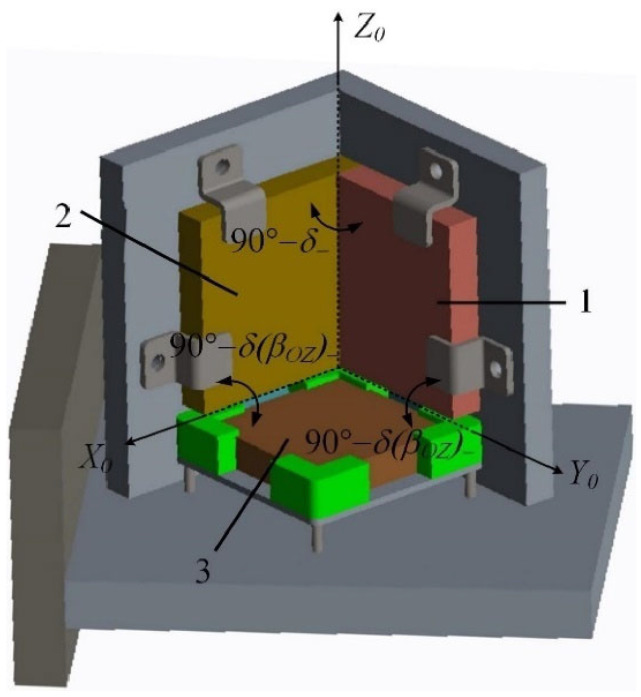
Hollow cylindrical cube-corner reflector (HCCCR) [247].

On the basis of high-precision, multi-degree-of-freedom measurement, there are currently few solutions to achieve a large measurement range. The main method is the autocollimator. Through the design of the measurement target shown in Figure 30, the measurement range can be expanded [247]. The experimental results show that the measuring range of the autocollimation angle measurement method is extended from ±30′ to ±30°, and the dynamic measurement distance is 0.2–5 m; the measurement accuracy of the pitch angle and the yaw angle is better than 69″ and 51″, respectively. This measurement solution can achieve two-dimensional angle measurement. It is believed that, in the future, by designing the structure of the grating, large-scale measurement of three-dimensional angles can also be achieved.

There are also some combined methods, that is, multi-degree-of-freedom angle measurement methods where multiple sensors work together.

**Figure 31:**
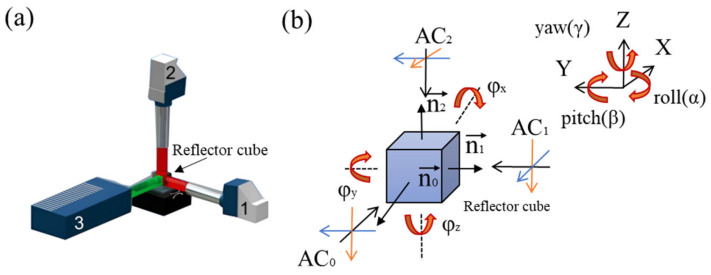
(**a**) Cartesian arrangement of the reference autocollimators (1—horizontal, 2—vertical) and the autocollimator (3) to be calibrated. (**b**) Schematic view of the SAAC.

Physikalisch Technische Bundesanstalt (PTB) created dedicated calibration devices that can follow different measurement principles and accomplish the task of metrological traceability in different ways [248]. For example, the Spatial Angle Autocollimator Calibrator (SAAC) is an advanced calibration device designed by PTB [219,249]. The system employs a novel Cartesian configuration consisting of three autocollimators (two reference autocollimators and one autocollimator) to precisely determine the angular position of the reflector cube, as shown in Figure 31. Each reference autocollimator is specifically designed to detect changes in one of the two corresponding tilt angles, while the autocollimator undergoing calibration is capable of sensing both angles. The distance between the reflector cube and the autocollimator being calibrated can be easily adjusted for flexibility.

**Figure 32:**
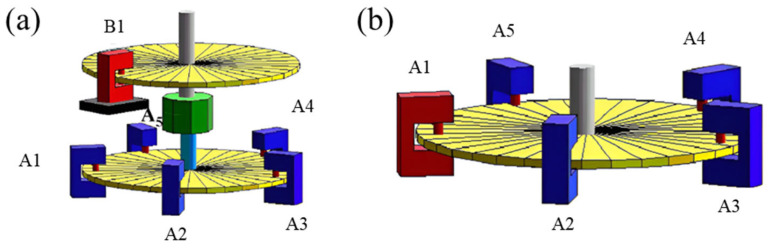
(**a**) Setup of two-rotary-encoder self-calibration system. (**b**) Setup of self-calibrating rotary encoder. The scale disks of two rotary encoders are A and B.

The National Metrology Institute of Japan (NMIJ) developed a rotary encoder calibration system to provide a highly precise standard calibration service [250], as shown in Figure 32. The new rotary encoder can calibrate itself and is composed of some reading heads that are arranged at equal angle intervals around a scale disk. This epoch-making rotary encoder is set up based on the national primary standard and compares these self-calibration data with the data using the primary standard system. The new rotary encoder can achieve an accuracy up to 0.5″ or less.

The National Institute of Standards and Technology (NIST) makes use of an automated stack of three indexing tables, namely the Advanced Automated Master Angle Calibration System (AAMACS) in conjunction with one of two possible instruments for small-angle measurement [251]. The proposed small-angle measurement system is a Fizeau phase-stepping interferometer rather than an autocollimator, which can avoid a variety of potential errors.

Laser dynamic goniometers are also devices that use lasers to measure angles, but are different from traditional laser gyroscopes. It usually uses a variety of sensors to work together, such as a ring laser and an optical angle sensor with the holographic principle of angular scale recording. It is a composite angle measurement method [252] that can determine the angle change and attitude information of the object with higher accuracy and stability.

**Figure 33:**
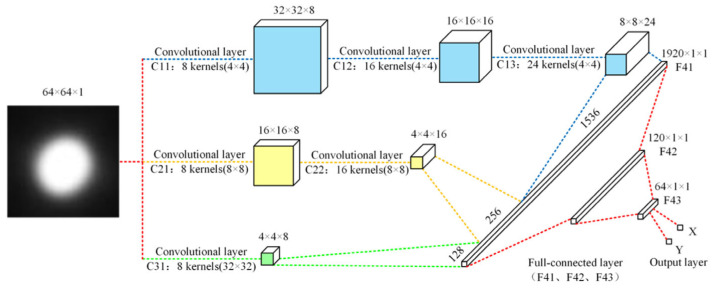
High-precision autocollimation method based on multiscale convolution neural network (MSCNN) [253].

Machine learning is a relatively novel method in high-accuracy measurement. It can use AI learning to learn the changing rules of physical phenomena that change with the measurement results during the measurement process, so that it can only observe different physical phenomena but directly output the measurement results without theoretical processes. Tan et al. proposed a high-precision autocollimation method based on a multiscale convolution neural network (MSCNN) for angle measurement, shown in Figure 33 [253]. Now, there are also schemes for deep learning measurement using CNN networks.

Table 2 shows the highest performance of the optical method with the measurement range, resolution, accuracy, and cost.

## 4. Conclusions and Prospects

This review briefly introduces various methods for angle measurement from both single-axis and multi-axis aspects. The aim of angle measurement schemes is to achieve high precision, multiple degrees of freedom, and a large measurement range.

The development of electrical and mechanical angle measuring instruments is relatively mature, and the cost of measurement solutions is low. However, the accuracy of electrical instruments is easily affected by environmental factors and most mechanical measuring instruments are contact measurements, which are limited in many applications. With the development of optical instruments and equipment, they perform better and have greater application prospects in high-precision measurement areas.

The autocollimator methods combined with measuring targets mainly detect the beam deflection to calculate angle information, and have the advantages of simple principles, non-contact measurement, and high accuracy. But they also have some limitations; for example, the laser autocollimator uses the laser wavelength as the measurement reference, which requires high frequency stability of the laser. The overall structure is relatively complex and there will be Abbe errors when measuring with multiple degrees of freedom. The grating encoder based on autocollimator methods uses the grating pitch as the measurement reference and has a more compact structure, but it also requires high frequency stability of the laser, the cost of optical components is high, and it has high requirements for the processing quality of gratings. The optical frequency comb has the advantages of high resolution and a wide measurement range, but it is sensitive to the environment factors, the overall system is very complex, and the working distance is limited. AI can also be used to detect the beam deflection to assist the angle measurement, which can use statistical methods to provide physical model relationships, which are difficult to give analytical expressions with. But it cannot be traced back to the source and measurement errors cannot be corrected, which is more suitable for measurement applications where the accuracy is not high.

In terms of high-precision measurement of angles, it is currently developing towards multiple degrees of freedom and higher-precision measurement. The self-designed targets currently used can achieve two-dimensional measurements of a larger angle range, while the angle measurement method of autocollimators is more universal. The three-dimensional angle measurement of the grating encoder based on the autocollimator method has been realized, and its target grating can also be used as a target for displacement measurement, making it easy to integrate. However, when implementing multiple-degree-of-freedom measurements, it is necessary to consider the coupling relationship between different degrees of freedom and the effect of coupling errors on the measurement. It is believed that in the future, it will also be a trend to implement a six-degree-of-freedom grating measurement solution that can match large angles through special structural designs for targets such as gratings.

## Figures and Tables

**Table 1:** The highest performance of the OFC method.

Parameters	Measurement Range	Resolution/Arcsec	Cost
Dispersive Characteristics of Grating [173]	>6°	0.03	middle
Absolute Distance Measurement [130]	>300 arcsec	0.073	Middle~high
The other OFC method [204]	>3.3°	0.23	Middle~high

**Table 2:** The highest performance of the optical method.

Parameters	Measurement Range	Resolution/Arcsec	Accuracy/Arcsec	Cost
Optical internal reflection	3 arcmin/−5.6–5°	0.02/0.288	0.02	low
Autocollimator	<30°	0.001 [227]	0.004 [227]	Middle~high
Visual method	5760 arcsec	1	3.6	middle
Phase method	Sub-degree	0.0007 [116].	0.695 [116].	high

## Data Availability

The data presented in this study are available on request from the corresponding author.

## References

[1] Kumar A.S.A., George B., Mukhopadhyay S.C. (2021). Technologies and Applications of Angle Sensors: A Review. IEEE Sens. J..

[2] Gao W., Kim S.W., Bosse H., Haitjema H., Chena Y.L., Lu X.D., Knapp W., Weckenmann A., Estler W.T., Kunzmann H. (2015). Measurement technologies for precision positioning. CIRP Ann.–Manuf. Technol..

[3] Zhang C., Liu W., Duan F., Fu X., Wang X. (2022). Accuracy improvement method and optimal design of straightness in five-degree-of-freedom measurement of long guide. Opt. Precis. Eng..

[4] Masuda T., Watanabe T., Beeks K., Fujimoto H., Hiraki T., Kaino H., Kitao S., Miyamoto Y., Okai K., Sasao N. (2021). Absolute X-ray energy measurement using a high-accuracy angle encoder. J. Synchrotron Radiat..

[5] Quan L., Shimizu Y., Xiong X., Matsukuma H., Gao W. (2021). A new method for evaluation of the pitch deviation of a linear scale grating by an optical angle sensor. Precis. Eng. J. Int. Soc. Precis. Eng. Nanotechnol..

[6] Chiu M.-H., Su D.-C. (1997). Angle measurement using total-internal-reflection heterodyne interferometry. Opt. Eng..

[7] Joo K.-N., Kim S.-W. (2006). Absolute distance measurement by dispersive interferometry using a femtosecond pulse laser. Opt. Express.

[8] Wu H., Zhang F., Liu T., Li J., Qu X. (2016). Absolute distance measurement with correction of air refractive index by using two-color dispersive interferometry. Opt. Express.

[9] Liang X., Lin J., Yang L., Wu T., Liu Y., Zhu J. (2020). Simultaneous Measurement of Absolute Distance and Angle Based on Dispersive Interferometry. IEEE Photonics Technol. Lett..

[10] Straube G., Fischer Calderón J.S., Ortlepp I., Füßl R., Manske E. (2021). A Heterodyne Interferometer with Separated Beam Paths for High-Precision Displacement and Angular Measurements. Nanomanuf. Metrol..

[11] Udem T., Holzwarth R., Hänsch T.W. (2002). Optical frequency metrology. Nature.

[12] Zhou S., Shen X., Wu C., Li D. The Error Analysis and Experimental Research of Micro-angle Measurement System Based on F-P Etalon. Proceedings of the 10th International Symposium on Precision Mechanical Measurements.

[13] Zelin S. Calibration method for measuring angle device with CCD. Proceedings of the 2021 International Conference of Optical Imaging and Measurement (ICOIM).

[14] Ren Y., Zhang F., Liu F., Hu X., Fu Y. (2022). Accuracy test method of multi-instrument cooperative measurement. Proc. SPIE.

[15] Wang S.T., Liao B.Q., Shi N.N., Li X.H. (2023). A Compact and High-Precision Three-Degree-of-Freedom Grating Encoder Based on a Quadrangular Frustum Pyramid Prism. Sensors.

[16] Kangning Y., Junhao Z., Weihan Y., Qian Z., Gaopeng X., Guanhao W., Xiaohao W., Xinghui L. (2021). Two-channel six degrees of freedom grating-encoder for precision-positioning of sub-components in synthetic-aperture optics. Opt. Express.

[17] Li X.H., Gao W., Muto H.S., Shimizu Y., Ito S., Dian S. (2013). A six-degree-of-freedom surface encoder for precision positioning of a planar motion stage. Precis. Eng. J. Int. Soc. Precis. Eng. Nanotechnol..

[18] Zhu J., Wang G., Wang S., Li X. (2022). A Reflective-Type Heterodyne Grating Interferometer for Three-Degree-of-Freedom Subnanometer Measurement. IEEE Trans. Instrum. Meas..

[19] Li X.H., Shimizu Y., Ito T., Cai Y.D., Ito S., Gao W. (2014). Measurement of six-degree-of-freedom planar motions by using a multiprobe surface encoder. Opt. Eng..

[20] Li X.H., Shi Y.P., Xiao X., Zhou Q., Wu G.H., Lu H.O., Ni K. (2018). Design and Testing of a Compact Optical Prism Module for Multi-Degree-of-Freedom Grating Interferometry Application. Appl. Sci..

[21] Wang G., Gao L., Huang G., Lei X., Cui C., Wang S., Yang M., Zhu J., Yan S., Li X. (2024). A wavelength-stabilized and quasi-common-path heterodyne grating interferometer with sub-nanometer precision. IEEE Trans. Instrum. Meas..

[22] Kim J.-A., Lee J.Y., Kang C.-S., Woo J.H. (2022). Hybrid optical measurement system for evaluation of precision two-dimensional planar stages. Measurement.

[23] Shimizu Y., Chen L.-C., Kim D.W., Chen X., Li X., Matsukuma H. (2020). An insight into optical metrology in manufacturing. Meas. Sci. Technol..

[24] Tamir B., Cohen E. (2013). Introduction to Weak Measurements and Weak Values. Quanta.

[25] Mi M., Malakhov M. (1970). Measuring Tilt Angle of Gear Teeth with an Optical Dividing Head. Meas. Tech..

[26] Bručas D., Giniotis V., Augustinavičius G., Stepanovienė J. (2010). Calibration of the multiangular prism (polygon). Mechanics.

[27] Lu X.D., Graetz R., Amin-Shahidi D., Smeds K. (2010). On-axis self-calibration of angle encoders. CIRP Ann..

[28] Liu X., Yu Z., Peng K., Zheng F., Pu H. (2022). Absolute Time-Grating Angular Displacement Sensor Based on Alternating Electric Field. The United Kingdom of Great Britain and Northern Ireland Patent.

[29] Liu X., Yu Z., Peng K., Zheng F., Pu H. (2021). Alternating Electric Field-Based Absolute Time-Grating Angular Displacement Sensor. Europe Patent.

[30] Liu X.K., Huang R., Yu Z.C., Peng K., Pu H.J. (2021). A High-Accuracy Capacitive Absolute Time-Grating Linear Displacement Sensor Based on a Multi-Stage Composite Method. IEEE Sens. J..

[31] Yu Z., Peng K., Liu X., Chen Z., Huang Y. (2019). A High-Precision Absolute Angular-Displacement Capacitive Sensor Using Three-Stage Time-Grating in Conjunction With a Remodulation Scheme. IEEE Trans. Ind. Electron..

[32] Kokuyama W., Watanabe T., Nozato H., Ota A. Measurement of Angle Error of Gyroscopes Using a Rotary Table Enhanced by Self-calibratable Rotary Encoder. Proceedings of the 2015 IEEE International Symposium on Inertial Sensors and Systems (ISISS) Proceedings.

[33] Benammar M., Ben-Brahim L., Alhamadi M.A. (2004). A Novel Resolver-to-360/Spl Deg/ Linearized Converter. IEEE Sens. J..

[34] Eggenstein F., Schwarz L., Zeschke T., Viefhaus J. (2021). New UHV angle encoder for high resolution monochromators, a modern spare part for the Heidenhain UHV RON 905. Nucl. Instrum. Methods Phys. Res. Sect. A Accel. Spectrometers Detect. Assoc. Equip..

[35] Sun S.Z., Han Y., Zhang H., He Z.Y., Tang Q.F. (2022). A Novel Inductive Angular Displacement Sensor With Multi-Probe Symmetrical Structure. IEEE Sens. J..

[36] Guo Q., Wang X., Xu C. (2022). Design of high precision angle measuring instrument based on Kalman filter. J. Phys. Conf. Ser..

[37] Li G., Xue Z., Huang Y., Zhu W., Zou W. (2021). Indication error analysis and compensation of circular grating angle measurement system. Chin. J. Sci. Instrum..

[38] Xia Y., Wu Z., Huang M., Liu X., Mi L., Tang Q. (2021). An Improved Angle Calibration Method of a High-Precision Angle Comparator. Metrol. Meas. Syst..

[39] Lu Y., Zhu W., Huang Y., Xue Z. (2021). Development of Dynamic Measuring Circuit of Circular Grating Angle Based on FPGA. Instrum. Technol. Sens..

[40] Qu P., Yan W., Sun S., Zhu T. (2022). Design of grating data acquisition system for airborne displacement and angle measurement. IET Conf. Proc..

[41] Li Z., Li J., Han B., Tang Y., Yanf Y., Ma Y. (2021). Compensation Method of Circular Grating Angle Measurement Error. Instrum. Technol. Sens..

[42] Hsieh T.-H., Watanabe T., Hsu P.-E. (2022). Calibration of Rotary Encoders Using a Shift-Angle Method. Appl. Sci..

[43] Huang Y., Che S., Zheng G., Zhu W., Zhou Y., Xue Z. (2022). An Adaptive Filter for Subdivision of Circular Grating Signal of Angle Measurement. IEEE Trans. Instrum. Meas..

[44] Zhao G., Ye G., Wu Z., Xing H., Liu S., Fan X., Li Y., Wei P., Liu H. (2021). On-line angle self-correction strategy based on a cobweb-structured grating scale. Meas. Sci. Technol..

[45] Janson N., Abt M., Kuester B., Overmeyer L. (2023). Optimization of optical rotation angle measurement by improved encodings. Tech. Mess..

[46] Minghao G., Lu W., Fenghao Z. (2021). Research on High Precision Angle Measurement and Compensation Technology Based on Circular Grating. J. Phys. Conf. Ser..

[47] Das S., Chakraborty B. (2018). Design and Realization of an Optical Rotary Sensor. IEEE Sens. J..

[48] Ahmadi Tameh T., Sawan M., Kashyap R. (2016). Novel Analog Ratio-Metric Optical Rotary Encoder for Avionic Applications. IEEE Sens. J..

[49] Liu B., Wang Y., Liu J., Chen A. Self-calibration Method of Circular Grating Error in Rotary Inertial Navigation. Proceedings of the 2023 2nd International Symposium on Sensor Technology and Control (ISSTC).

[50] Du Y., Yuan F., Jiang Z., Li K., Yang S., Zhang Q., Zhang Y., Zhao H., Li Z., Wang S. (2021). Strategy to Decrease the Angle Measurement Error Introduced by the Use of Circular Grating in Dynamic Torque Calibration. Sensors.

[51] Chen Z., Liu X., Peng D., Zheng Y., Chen X., Zheng F. (2012). Dynamic model of NC rotary table in angle measurements with time series. Trans. Inst. Meas. Control.

[52] Zhou S., Le V., Mi Q., Wu G. (2020). Grating-Corner-Cube-Based Roll Angle Sensor. Sensors.

[53] Zhou S., Le V., Xiong S., Yang Y., Ni K., Zhou Q., Wu G. (2021). Dual-comb spectroscopy resolved three-degree-of-freedom sensing. Photonics Res..

[54] Du X., Chen Q. (2021). Dual-Laser Goniometer: A Flexible Optical Angular Sensor for Joint Angle Measurement. IEEE Trans. Ind. Electron..

[55] Zhao Y., Zha C., Liu B., Han F. (2023). High-precision rotation angle measurement method based on polarization self-mixing interference. Appl. Opt..

[56] Zhao Y., Zhang H. (2021). Angle measurement method based on speckle affected laser self-mixing interference signal. Opt. Commun..

[57] Zhao Y., Xiang R., Chen J., Huang Z., Wang X., Ma Y., Yu B., Lu L. (2021). Absolute angle-measurement with a dihedral mirror of the multi-longitudinal mode laser self-mixing sensor. Precis. Eng. J. Int. Soc. Precis. Eng. Nanotechnol..

[58] Yu H., Jia X., Wan Q., Liang L., Zhao C. (2019). High-Resolution Angular Displacement Technology Based on Varying Moiré Figure Phase Positions. IEEE Sens. J..

[59] Chen G. (2020). Improving the angle measurement accuracy of circular grating. Rev. Sci. Instrum..

[60] Lee C.H., Huang H.J., Chang J.P., Chen Y.C. (2022). Incremental Optical Encoder Based on a Sinusoidal Transmissive Pattern. IEEE Photonics J..

[61] Kato Y., Mizutani M. (2022). Photoelectric rotary encoder. U.S. Patent.

[62] Dong L., Ruan Y., Wang J., Wang B., Li H., Guo P., Wu Y., Jiang S., Zhang S., Wei P. (2021). Progress in High Accurate Angle Measurement Technology of Long-Distance Target Based on Computational Interferometry. Laser Optoelectron. Prog..

[63] Huang P.S., Kiyono S., Kamada O. (1992). Angle measurement based on the internal-reflection effect: A new method. Appl. Opt..

[64] Zheng F., Long F., Zhao Y., Yu C., Jia P., Zhang B., Yuan D., Feng Q. (2023). High-Precision Small-Angle Measurement of Laser-Fiber Autocollimation Using Common-Path Polarized Light Difference. IEEE Sens. J..

[65] Mandal L., Singh J., Ganesan A.R. (2023). Michelson Interferometer with mirrored right-angled prism for measurement of tilt with double sensitivity. J. Opt..

[66] Kong Q., Wen L. (2022). Fast tilt measurement based on optical block. Opt. Laser Technol..

[67] Pisani M., Astrua M. (2006). Angle amplification for nanoradian measurements. Appl. Opt..

[68] Liu X., Wang Y., Zhu L., Wang B., Yan P. (2023). High-precision dynamic measurement of roll angle based on digital holography. Opt. Lasers Eng..

[69] Pang J., Tan J., Niu Y., Liu Y., Pan W. (2022). Angular displacement measurement method based on least square fitting wave vector estimation. Opt. Technol..

[70] Chen H., Chen Q., Chen H., Yang X., Wang X. (2023). Measurement of displacement and top beam attitude angle of advanced hydraulic support based on visual detection. Measurement.

[71] Liu H., Shen Y., Liang L., Chen P., Yu K., Wang K. Angle measurement method based on axicon lens. Proceedings of the Conference on AOPC—Infrared Device and Infrared Technology.

[72] Mo L., Ren J., Ren M. (2022). Microlens Array-Based Spatial Angle Encoding for High-Precision Visual Pose Measurement. Acta Opt. Sin..

[73] Liu H., Zhao S., Liu J., Chen P., Zhang L., Wang K. (2023). Non-imaging method for angle measurement based on an axicon. Appl. Opt..

[74] Grubert N., Koenig G., Stollenwerk J., Loosen P. Ring-Shaped Conoscopic Holography for Distance and Tilt Measurement. Proceedings of the Conference on Lasers and Electro-Optics (CLEO), Electr Network.

[75] Kim H., Yamakawa Y., Senoo T., Ishikawa M. (2016). Visual encoder: Robust and precise measurement method of rotation angle via high-speed RGB vision. Opt. Express.

[76] Cheng H., Wang Y., Wei K., Liu Z., Yang M., Cai C. (2022). Visual encoder-based angle measurement method in low-frequency angular vibration calibration. Appl. Opt..

[77] Li Z., Pei Y., Qu C., Yang F. (2022). A Position and Attitude Measurement Method Based on Laser Displacement Sensor and Infrared Vision Camera. IEEE Trans. Instrum. Meas..

[78] Wei Z., Feng G., Zhou D., Ma Y., Liu M., Luo Q., Huang T. (2023). A Review of Position and Orientation Visual Measurement Methods and Applications. Laser Optoelectron. Prog..

[79] Lowe D.G. (2004). Distinctive Image Features from Scale-Invariant Keypoints. Int. J. Comput. Vis..

[80] Xue N., Bai S., Wang F., Xia G.-S., Wu T., Zhang L. Learning Attraction Field Representation for Robust Line Segment Detection. Proceedings of the 2019 IEEE/CVF Conference on Computer Vision and Pattern Recognition (CVPR).

[81] Von Gioi R.G., Jakubowicz J., Morel J.M., Randall G. (2010). LSD: A Fast Line Segment Detector with a False Detection Control. IEEE Trans. Pattern Anal. Mach. Intell..

[82] Hodan T., Barath D., Matas J. EPOS: Estimating 6D Pose of Objects With Symmetries. Proceedings of the 2020 IEEE/CVF Conference on Computer Vision and Pattern Recognition (CVPR).

[83] Choi C., Christensen H.I. Real-time 3D Model-based Tracking Using Edge and Keypoint Features for Robotic Manipulation. Proceedings of the 2010 IEEE International Conference on Robotics and Automation.

[84] Choi C., Christensen H.I. Robust 3D visual tracking using particle filtering on the SE(3) group. Proceedings of the 2011 IEEE International Conference on Robotics and Automation.

[85] Xiong S., Wang Y., Cai Y., Liu J., Liu J., Wu G. (2018). Calculating the Effective Center Wavelength for Heterodyne Interferometry of an Optical Frequency Comb. Appl. Sci..

[86] Kleinman D.A., Ashkin A., Boyd G.D. (1966). Second-harmonic generation of light by focused laser beams. Phys. Rev..

[87] Dwi Astuti W., Matsukuma H., Nakao M., Li K., Shimizu Y., Gao W. (2021). An Optical Frequency Domain Angle Measurement Method Based on Second Harmonic Generation. Sensors.

[88] Aharonov Y., Albert D.Z., Vaidman L. (1988). How the Result of a Measurement of a Component of the Spin of a Spin-1/2 Particle Can Turn out to Be 100. Phys. Rev. Lett..

[89] Dixon P.B., Starling D.J., Jordan A.N., Howell J.C. (2009). Ultrasensitive Beam Deflection Measurement via Interferometric Weak Value Amplification. Phys. Rev. Lett..

[90] Dressel J., Malik M., Miatto F.M., Jordan A.N., Boyd R.W. (2014). Understanding quantum weak values: Basics and applications. Rev. Mod. Phys..

[91] Wang Y.T., Tang J.S., Hu G., Wang J., Yu S., Zhou Z.Q., Cheng Z.D., Xu J.S., Fang S.Z., Wu Q.L. (2016). Experimental Demonstration of Higher Precision Weak-Value-Based Metrology Using Power Recycling. Phys. Rev. Lett..

[92] Qiu X.D., Xie L.G., Liu X., Luo L., Li Z.X., Zhang Z.Y., Du J.L. (2017). Precision phase estimation based on weak-value amplification. Appl. Phys. Lett..

[93] Xia B.K., Huang J.Z., Li H.J., Wang H., Zeng G.H. (2023). Toward incompatible quantum limits on multiparameter estimation. Nat. Commun..

[94] Zhang Y., Liu X., Zhang T., Xu Y., Wang Y., Wang Z. (2023). Micro-angle attitude measurement technology and research progress based on quantum weak value measurement. Proc. SPIE.

[95] Cherepanska I.Y., Sazonov A.Y., Krushynska N.I., Priadko V.A., Lukiniuk M.V. (2021). Quaternion Method of Calculating Angles while Measuring via Goniometric Precision Instrument System. Bull. Univ. Karaganda-Phys..

[96] Ahmed A., Ju H.H., Yang Y., Xu H. (2023). An Improved Unit Quaternion for Attitude Alignment and Inverse Kinematic Solution of the Robot Arm Wrist. Machines.

[97] Wang X., Su J., Yang J., Miao L., Huang T. (2021). Investigation of heterodyne interferometer technique for dynamic angle measurement: Error analysis and performance evaluation. Meas. Sci. Technol..

[98] Mokros J. (1990). Angle-measuring interferometer. Optoelectron. Instrum. Data Process..

[99] Crane R. (1979). Angle-Scanned Interferometer. Opt. Eng..

[100] Dai X., Sasaki O., Greivenkamp J.E., Suzuki T. (1997). High accuracy, wide range, rotation angle measurement by the use of two parallel interference patterns. Appl. Opt..

[101] Tomlinson W.J., Mollenauer L.F. (1977). Balanced Interferometers with Simply Adjustable Interference Angle. Appl. Opt..

[102] Khoroshun A.N., Artsishevskii D.N. (2010). Determining small angles of beam splitter rotation in optical vortex shearing interferometer. Tech. Phys. Lett..

[103] Donati S., Norgia M. Displacement and attitude angles (Tilt and Yaw) are measured by a single-channel self-mixing interferometer. Proceedings of the CLEO: Applications and Technology 2016.

[104] Serrano Galvez P., Butcher M., Masi A. (2019). A Dual-Interferometer-Based Angular Measurement System With Absolute Angle Recovery Method. IEEE Trans. Instrum. Meas..

[105] Liang X., Lin J., Yang L., Wu T., Liu Y., Zhu J. Femtosecond pulse laser distance and angle interferometer. Proceedings of the International Conference on Optical Instruments and Technology (OIT)—Optoelectronic Measurement Technology and Systems.

[106] Shestopalov Y.N. (1990). Interference Methods and Means for Measuring Angles. Sov. J. Opt. Technol..

[107] Takashima K. (1969). A Modified Angle Measuring Interferometer. Jpn. J. Appl. Phys..

[108] Jiapeng L., Yi W., Zunyue Z., Yaojing Z., Hon Ki T. Multimode Wavelength Division Demultiplexing Based on an Angled Multimode Interference Coupler. Proceedings of the 2021 Asia Communications and Photonics Conference (ACP) 2021.

[109] Wang G.-F., Chen G.-L., Chen Y.-L. (2001). A new digital angle measurement technology by using Michelson interferometer and its application. Opt. Precis. Eng..

[110] Shi P., Stijns E. (1988). New Optical Method for Measuring Small-Angle Rotations. Appl. Opt..

[111] Chekirda K.V., Shur V.L., Lukin A.Y., Kos’mina M.A., Leibengardt G.I. (2016). A Study of an Angle Examiner Based on the Fizeau Interferometer with Expanded Measurement Range. Meas. Tech..

[112] Wang X., Yang J., Huang T., Shu X. A high-frequency angular vibration calibration system based on phase modulated laser interferometer technique. Proceedings of the Conference on AOPC—Micro-Optics and MOEMS.

[113] Eves B.J., Leroux I.D., Cen A.J. (2023). Phase shifting angle interferometer. Metrologia.

[114] Twu R.-C., Lin B.-L. (2022). Development and investigation of birefringent Beta-Barium borate for optical angle sensor. Opt. Laser Technol..

[115] Chen S.-H., Chen H.-Y., Lai Y.-X., Hsieh H.-L. Development of a double-diffraction grating interferometer for measurements of displacement and angle. Proceedings of the Conference on Optics and Photonics for Advanced Dimensional Metrology, Electr Network.

[116] Hsu Y.-T., Hsieh H.-L. Development of a Coplanar Grating Interferometer for Displacement and Angle Measurements. Proceedings of the Conference on Optics and Photonics for Advanced Dimensional Metrology II Part of SPIE Photonics Europe Conference, Electr Network.

[117] Xu X., Dai Z.R., Wang Y.F., Li M.F., Tan Y.D. (2022). High Sensitivity and Full-Circle Optical Rotary Sensor for Non-Cooperatively Tracing Wrist Tremor With Nanoradian Resolution. IEEE Trans. Ind. Electron..

[118] Xu X., Dai Z., Tan Y. (2023). A Dual-Beam Differential Method Based on Feedback Interferometry for Noncontact Measurement of Linear and Angular Displacement. IEEE Trans. Ind. Electron..

[119] Yu L., Feng X., Hu P., Lin X., Jing T. (2023). Development of a 3-DOF Angle Sensor Based on a Single Laser Interference Probe. Micromachines.

[120] Shi K., Su J., Hou W. (2018). Roll angle measurement system based on differential plane mirror interferometer. Opt. Express.

[121] Liang X., Lin J., Wu T., Yang L., Wang Y., Liu Y., Zhu J. (2020). Absolute angular measurement with optical frequency comb using a dispersive interferometry. Opt. Express.

[122] Liu T., Wu J., Suzuki A., Sato R., Matsukuma H., Gao W. (2023). Improved Algorithms of Data Processing for Dispersive Interferometry using Optical Frequency Comb. Preprints.

[123] Hargrove L.E., Fork R.L., Pollack M.A. (1964). Locking of He–Ne Laser Modes Induced by Synchronous Intracavity Modulation. Appl. Phys. Lett..

[124] Chang L., Liu S., Bowers J.E. (2022). Integrated optical frequency comb technologies. Nat. Photonics.

[125] Shimizu Y., Matsukuma H., Gao W. (2020). Optical Angle Sensor Technology Based on the Optical Frequency Comb Laser. Appl. Sci..

[126] Fortier T., Baumann E. (2019). 20 years of developments in optical frequency comb technology and applications. Commun. Phys..

[127] Diddams S.A. (2010). The evolving optical frequency comb. J. Opt. Soc. Am. B.

[128] Newbury N.R. (2011). Searching for applications with a fine-tooth comb. Nat. Photonics.

[129] Diddams S.A., Vahala K., Udem T. (2020). Optical frequency combs: Coherently uniting the electromagnetic spectrum. Science.

[130] Han S., Kim Y.-J., Kim S.-W. (2015). Parallel determination of absolute distances to multiple targets by time-of-flight measurement using femtosecond light pulses. Opt. Express.

[131] Dale J., Hughes B., Lancaster A.J., Lewis A.J., Reichold A.J.H., Warden M.S. (2014). Multi-channel absolute distance measurement system with sub ppm-accuracy and 20 m range using frequency scanning interferometry and gas absorption cells. Opt. Express.

[132] Cao K., Qiu C., Gong Y., Li J. (2023). An accurate measurement method of gear transmission error based on a double-angle measurement device. Proc. Inst. Mech. Eng. Part C J. Mech. Eng. Sci..

[133] Li Y., Shen T., Zhang Y., Zhao L., Zhou L. (2021). The Five-axis Turn-table Angle Location Error Analysis based on Dual-frequency Laser Phase Measure System. J. Astronaut. Metrol. Meas..

[134] Andevari S.A., Olvera-Cervantes J.-L., Saavedra C.E. (2023). Inclination Sensor With a Wide Angle Measurement Range Using Half-Wavelength Microstrip Resonator. IEEE Access.

[135] Wu L., Su R., Tong P., Yanchen A., Wu Y. (2023). An inductive sensor for the angular displacement measurement of large and hollow rotary machinery. IEEE Sens. J..

[136] Komatsuzaki S., Takeyama A., Sado K., Nagatsu Y., Hashimoto H. Absolute Angle Calculation for Magnetic Encoder Based On Magnetic Flux Density Difference. Proceedings of the 47th Annual Conference of the IEEE-Industrial-Electronics-Society (IECON), Electr Network.

[137] Jeong S.-H., Rhyu S.-H., Kwon B.-I., Kim B.-T. (2007). Design of the Rotary Magnetic Position Sensor With the Sinusoidally Magnetized Permanent Magnet. IEEE Trans. Magn..

[138] Zhang Z., Ni F., Dong Y., Guo C., Jin M., Liu H. (2015). A Novel Absolute Magnetic Rotary Sensor. IEEE Trans. Ind. Electron..

[139] Wu S.-T., Chen J.-Y., Wu S.-H. (2014). A Rotary Encoder With an Eccentrically Mounted Ring Magnet. IEEE Trans. Instrum. Meas..

[140] Liu C., Xu F. A Novel Absolute Angle-Measuring Method with Rotary Inductosyn. Proceedings of the 2018 Eighth International Conference on Instrumentation & Measurement, Computer, Communication and Control (IMCCC).

[141] Zhang Z., Ni F., Dong Y., Jin M., Liu H. (2013). A novel absolute angular position sensor based on electromagnetism. Sens. Actuators A Phys..

[142] Dezhi Z., Shaobo Z., Shuai W., Chun H., Xiaomeng Z. (2015). A Capacitive Rotary Encoder Based on Quadrature Modulation and Demodulation. IEEE Trans. Instrum. Meas..

[143] Anandan N., George B. (2017). A Wide-Range Capacitive Sensor for Linear and Angular Displacement Measurement. IEEE Trans. Ind. Electron..

[144] Sun S.Z., Lv Z., Han Y., He Z.Y., Zhang J.M. (2022). A novel inductive angular displacement sensor based on time-grating. Meas. Sci. Technol..

[145] Chen X., He X., Zhang Y., Gao B. Angle Measurement Method Based on Cross-Orthogonal Hall Sensor. Proceedings of the 2023 IEEE 16th International Conference on Electronic Measurement & Instruments (ICEMI).

[146] Dorofeev N.V., Kuzichkin O.R., Tsaplev A.V. (2015). Accelerometric Method of Measuring the Angle of Rotation of the Kinematic Mechanisms of Nodes. Appl. Mech. Mater..

[147] Gao Y., Zhang X., Mai G., Hu R. (2015). Measurement Method for Angle Acceleration of Turn-table Based on Accelerometer. J. Astronaut. Metrol. Meas..

[148] Lu Y.-l., Pan Y.-J., Li L.-l., Liu Y., Peng H. (2015). Measurement method of projectile’s heading and pitching angle velocities based on biaxial accelerometer. J. Chin. Inert. Technol..

[149] Shi K., Xu G., Qian R., Wang J. (2016). Single Accelerometer Roll Angle Measurement System Error Analysis. J. Detect. Control.

[150] Song H., Huang H., Zhang J., Qu D. (2018). A new measurement-while-drilling system based on inertial technology. Adv. Mech. Eng..

[151] Tian H., Liu Y., Zhou J., Wang Y., Wang J., Zhang W. (2019). Attitude Angle Compensation for a Synchronous Acquisition Method Based on an MEMS Sensor. Sensors.

[152] Wang K., Wu Y. (2022). Error Compensation Technology for Inclinometer Accelerometer Bias. Instrum. Technol. Sens..

[153] Jabbar K.A., Khemri N.A., Brayyich M.R., Ftaiet A.A. (2023). Design of a tilt angle measurement and control system. AIP Conf. Proc..

[154] Ren C., Guo D., Zhang L., Wang T. (2022). Research on Nonlinear Compensation of the MEMS Gyroscope under Tiny Angular Velocity. Sensors.

[155] Lijia Y., Qiang L., Guochen W., Hualiang S., Qingzhi Z. (2022). An on-orbit high-precision angular vibration measuring device based on laser gyro. Proc. SPIE.

[156] Liu Y., Luo X., Chen X. (2022). Improvement of angle random walk of fiber-optic gyroscope using polarization-maintaining fiber ring resonator. Opt. Express.

[157] Ma R., Yu H., Ni K., Zhou Q., Li X., Wu G. Angular velocity measurement system based on optical frequency comb. Proceedings of the Conference on Semiconductor Lasers and Applications IX.

[158] Yu C., Cai Y., Ren Y., Wang W., Yin Z., Li L., Han W., Han W. (2022). Angular Rate Measurement Method of Magnetically Suspended Control and Sensing Gyroscope Using Component-Level Rotation Modulation. IEEE Sens. J..

[159] Sugiyama Y., Matsui Y., Toyoda H., Mukozaka N., Ihori A., Abe T., Takabe M., Mizuno S. (2008). A 3.2 kHz, 14-Bit Optical Absolute Rotary Encoder With a CMOS Profile Sensor. IEEE Sens. J..

[160] Kim J.-A., Kim J.W., Kang C.-S., Jin J., Eom T.B. (2016). Absolute angle measurement using a phase-encoded binary graduated disk. Measurement.

[161] Yu H., Jia X., Wan Q., Zhao C., Sun Y. (2020). High-resolution angular measurement arithmetic based on pixel interpolations. Measurement.

[162] Kim J.-A., Lee J.Y., Kang C.-S., Eom S.H. (2023). Measurement of Six-Degree-of-Freedom Absolute Postures Using a Phase-Encoded Pattern Target and a Monocular Vision System. Int. J. Precis. Eng. Manuf..

[163] Dong H., Fu Q., Zhao X., Quan Q., Zhang R. (2015). Practical rotation angle measurement method by monocular vision. Appl. Opt..

[164] Yu H. (2021). Angle measurement based on in-line digital holographic reconstruction. Opt. Lasers Eng..

[165] Gao S., Bai L. (2021). Monocular Camera-Based Three-Point Laser Pointer Ranging and Pose Estimation Method. Acta Opt. Sin..

[166] Zhao Y., Zhang Y., Zhang H., Xue L., Ren M., Miao Y. (2022). Rotation angle measurement method based on self-mixing interference of a fiber laser. Appl. Opt..

[167] Vadapalli D.R.P., Dey K., Roy S. (2021). Optical Fiber-Based Intensity-Modulated Cost-Effective Small Lean Angle Measurement Sensor. Mapan-J. Metrol. Soc. India.

[168] Zheng L.-F., Zhang J.-S., Liang H.-J., Wang H.-J. (2022). Angle sensor for humidity-insensitive angle measurement based on multimode interference. Opt. Commun..

[169] Dai M., Zhang C., Pan X., Yang Y., Li Z. (2022). A Novel Attitude Measurement While Drilling System Based on Single-Axis Fiber Optic Gyroscope. IEEE Trans. Instrum. Meas..

[170] Yusuke S., Yoshikazu A., Wei G. (2009). Detection of three-axis angles by an optical sensor. Sens. Actuators A Phys..

[171] Heikkinen V., Byman V., Palosuo I., Hemming B., Lassila A. (2017). Interferometric 2D small angle generator for autocollimator calibration. Metrologia.

[172] Gao W. (2019). Metrology.

[173] Chen Y.-L., Shimizu Y., Tamada J., Kudo Y., Madokoro S., Nakamura K., Gao W. (2017). Optical frequency domain angle measurement in a femtosecond laser autocollimator. Opt. Express.

[174] Chen Y.-L., Shimizu Y., Tamada J., Nakamura K., Matsukuma H., Chen X., Gao W. (2018). Laser autocollimation based on an optical frequency comb for absolute angular position measurement. Precis. Eng..

[175] Matsukuma H., Ikeda K., Sato R., Gao W. (2023). Autocollimation employing optical frequency comb. Proc. SPIE.

[176] Chen Y.-L., Shimizu Y., Kudo Y., Ito S., Gao W. (2016). Mode-locked laser autocollimator with an expanded measurement range. Opt. Express.

[177] Shimizu Y., Madokoro S., Matsukuma H., Gao W. (2018). An optical angle sensor based on chromatic dispersion with a mode-locked laser source. J. Adv. Mech. Des. Syst. Manuf..

[178] Gao W., Saito Y., Muto H., Arai Y., Shimizu Y. (2011). A three-axis autocollimator for detection of angular error motions of a precision stage. CIRP Ann.-Manuf. Technol..

[179] Wang S.T., Luo L.B., Zhu J.H., Shi N.N., Li X.H. (2022). An Ultra-Precision Absolute-Type Multi-Degree-of-Freedom Grating Encoder. Sensors.

[180] Li X.H., Gao W., Shimizu Y., Ito S. (2014). A two-axis Lloyd’s mirror interferometer for fabrication of two-dimensional diffraction gratings. CIRP Ann.-Manuf. Technol..

[181] Li X.H., Shimizu Y., Ito S., Gao W. (2013). Fabrication of Scale Gratings for Surface Encoders by Using Laser Interference Lithography with 405 nm Laser Diodes. Int. J. Precis. Eng. Manuf..

[182] Zhou Q., Pang J.C., Li X.H., Ni K., Tian R. (2015). Concave grating miniature spectrometer with an expanded spectral band by using two entrance slits. Chin. Opt. Lett..

[183] Xue G.P., Zhai Q.H., Lu H.O., Zhou Q., Ni K., Lin L.Y., Wang X.H., Li X.H. (2021). Polarized holographic lithography system for high-uniformity microscale patterning with periodic tunability. Microsyst. Nanoeng..

[184] Li X.H., Ni K., Zhou Q., Wang X.H., Tian R., Pang J.C. (2016). Fabrication of a concave grating with a large line spacing via a novel dual-beam interference lithography method. Opt. Express.

[185] Zhou Q., Li X.H., Ni K., Tian R., Pang J.C. (2016). Holographic fabrication of large-constant concave gratings for wide-range flat-field spectrometers with the addition of a concave lens. Opt. Express.

[186] Matsukuma H., Ishizuka R., Furuta M., Xinghui L., Shimizu Y., Wei G. (2019). Reduction in Cross-Talk Errors in a Six-Degree-of-Freedom Surface Encoder. Nanomanuf. Metrol..

[187] Xue G.P., Lu H.O., Li X.H., Zhou Q., Wu G.H., Wang X.H., Zhai Q.H., Ni K. (2020). Patterning nanoscale crossed grating with high uniformity by using two-axis Lloyd’s mirrors based interference lithography. Opt. Express.

[188] Li X.H., Zhou Q., Zhu X.W., Lu H.O., Yang L., Ma D.H., Sun J.H., Ni K., Wang X.H. (2017). Holographic fabrication of an arrayed one-axis scale grating for a two-probe optical linear encoder. Opt. Express.

[189] Li X.H., Lu H.O., Zhou Q., Wu G.H., Ni K., Wang X.H. (2018). An Orthogonal Type Two-Axis Lloyd’s Mirror for Holographic Fabrication of Two-Dimensional Planar Scale Gratings with Large Area. Appl. Sci..

[190] Xu B., Jia Z., Li X., Chen Y.-L., Shimizu Y., Ito S., Gao W. Surface form metrology of micro-optics. Proceedings of the International Conference on Optics in Precision Engineering and Nanotechnology, icOPEN 2013.

[191] Li X., Zhu X., Zhou Q., Wang H., Ni K. Low-cost lithography for fabrication of one-dimensional diffraction gratings by using laser diodes. Proceedings of the 2015 International Conference on Optical Instruments and Technology: Micro/Nano Photonics and Fabrication, OIT 2015.

[192] Li X.H., Ni K., Zhou Q., Yan P., Pang J.C., Wang X.H. (2017). Improved master-replica separation process for fabrication of a blazed concave grating by using a combination-type convex grating. Appl. Opt..

[193] Wang G.C., Xue G.P., Zhai Q.H., Zhu J.H., Yu K.N., Huang G.Y., Wang M., Zhong A.H., Zhu L.X., Yan S.H. (2021). Planar diffractive grating for magneto-optical trap application: Fabrication and testing. Appl. Opt..

[194] Gao W., Kimura A. (2007). A three-axis displacement sensor with nanometric resolution. CIRP Ann.-Manuf. Technol..

[195] Liao B., Wang S., Lin J., Dou Y., Wang X., Li X., Zhu J., Jiang J., Han S., Zeng L. A research on compact short-distance grating interferometer based on ridge prism. Proceedings of the 2021 International Conference on Optical Instruments and Technology: Optoelectronic Measurement Technology and Systems.

[196] Wang S., Gao L., Luo L., Deng F., Wang X., Ma R., Li X. Codes coupling optimization for absolute measurement. Proceedings of the Optical Metrology and Inspection for Industrial Applications X.

[197] Wang S., Luo L., Gao L., Wang X., Ma R., Li X. Long binary coding design for absolute positioning using genetic algorithm. Proceedings of the Optical Metrology and Inspection for Industrial Applications X.

[198] Wang S., Zhu J., Shi N., Luo L., Wen Y., Li X. Modeling and test of an absolute four-degree-of-freedom (DOF) grating encoder. Proceedings of the Optical Metrology and Inspection for Industrial Applications IX, Electr Network.

[199] Shimizu Y., Kudo Y., Chen Y.-L., Ito S., Gao W. (2017). An optical lever by using a mode-locked laser for angle measurement. Precis. Eng..

[200] Shan S., Li J., Liu P., Li Q., Wang X., Li X. (2023). A Microlens Array Grating for Miniature Multi-Channel Spectrometers. Sensors.

[201] Tan S.L., Shimizu Y., Meguro T., Ito S., Gao W. (2015). Design of a laser autocollimator-based optical sensor with a rangefinder for error correction of precision slide guideways. Int. J. Precis. Eng. Manuf..

[202] Shimizu Y., Tan S.L., Murata D., Maruyama T., Ito S., Chen Y.-L., Gao W. (2016). Ultra-sensitive angle sensor based on laser autocollimation for measurement of stage tilt motions. Opt. Express.

[203] Gao W., Ohnuma T., Satoh H., Shimizu H., Kiyono S. (2004). A Precision Angle Sensor Using a Multi-cell Photodiode Array. CIRP Ann..

[204] Matsukuma H., Madokoro S., Astuti W.D., Shimizu Y., Gao W. (2019). A New Optical Angle Measurement Method Based on Second Harmonic Generation with a Mode-Locked Femtosecond Laser. Nanomanuf. Metrol..

[205] Matsukuma H., Asumi Y., Nagaoka M., Shimizu Y., Gao W. (2021). An autocollimator with a mid-infrared laser for angular measurement of rough surfaces. Precis. Eng..

[206] Lim H., Shimizu Y. (2023). Feasible Resolution of Angular Displacement Measurement by an Optical Angle Sensor Based on Laser Autocollimation. Nanomanuf. Metrol..

[207] Astrua M., Pisani M. (2021). Improved performance of a refurbished photoelectric autocollimator. Meas. Sci. Technol..

[208] Luo L., Gao L., Wang S., Deng F., Wu Y., Li X. An ultra-precision error estimation for a multi-axes grating encoder using quadrant photodetectors. Proceedings of the Conference on Optical Metrology and Inspection for Industrial Applications IX Part of SPIE/COS Photonics Asia Conference, Electr Network.

[209] Gibson S.J., Charrett T.O.H., Tatam R.P., Lehmann P., Osten W., Albertazzi Gonçalves A. Absolute angle measurement using dual-wavelength laser speckle for robotic manufacturing. Proceedings of the Optical Measurement Systems for Industrial Inspection XI.

[210] Yan B., Tan Q., Lv N. Application of photoelectric autocollimator in detecting position precision of NC motorized stage. Proceedings of the Conference on Optical Metrology and Inspection for Industrial Applications.

[211] Yandayan T., Akgoz S.A., Asar M. (2014). Calibration of high-resolution electronic autocollimators with demanded low uncertainties using single reading head angle encoders. Meas. Sci. Technol..

[212] Peng L.I.U., Li-min G.A.O., Su-wen Z. (2010). CCD in the Autocollimator Used for Moire Fringe Imaging. Acta Photonica Sin..

[213] Sharma B.D. (1975). Design and testing of a multipurpose autocollimator. CSIO Commun..

[214] Yan B., Tan Q., Lv N. Detection of Positional Precision of NC Motorized Stage Based on Photoelectric Autocollimator. Proceedings of the 6th International Symposium on Precision Engineering Measurements and Instrumentation.

[215] Andreeva T.A., Bokhman E.D., Venediktov V.Y., Gordeev S.V., Korolev A.N., Kos’mina M.A., Lukin A.Y., Shur V.L. (2018). Estimation of metrological characteristics of a high-precision digital autocollimator using an angle encoder. J. Opt. Technol..

[216] Shen M.Z., Liao S. (2005). A high precision dynamic autocollimator. Meas. Technol. Intell. Instrum. VI.

[217] Heikkinen V., Byman V., Shpak M., Geckeler R., Just A., Krause M., Schumann M., Lassila A. High-accuracy autocollimator calibration by interferometric 2D angle generator. Proceedings of the Conference on Advances in Metrology for X-Ray and EUV Optics VIII.

[218] Bergues G.J., Schurrer C., Brambilla N., Canali L. Misalignment contribution to the autocollimator’s scale distortion. Proceedings of the IEEE International Instrumentation and Measurement Technology Conference (I2MTC), Politecnico Torino.

[219] Kranz O., Geckeler R.D., Just A., Krause M. Modelling PTB’s Spatial Angle Autocollimator Calibrator. Proceedings of the Conference on Modeling Aspects in Optical Metrology IV as part of the SPIE Optical Metrology Symposium.

[220] Wei Y., Wu Y., Xiao M., Lu W. Optical System Design of Dual-spectrum Autocollimator. Proceedings of the Annual Conference of the Chinese-Society-for-Optical-Engineering on Applied Optics and Photonics, China (AOPC).

[221] Gao Y., Liao J., Zhao X., Xu J., Shen X., Zhu H. Optimal design for three dimensional autocollimator. Proceedings of the Annual Conference of the Chinese-Society-for-Optical-Engineering (CSOE) on Applied Optics and Photonics China (AOPC)—3D Measurement Technology for Intelligent Manufacturing.

[222] Filatov Y.V., Larichev R.A. Precision issues in angle measurements by means of autocollimator. Proceedings of the Conference on Optical Metrology and Inspection for Industrial Applications III held as part of SPIE/COS Photonics Asia.

[223] Hu T., Wang D., Li Z. Research of the Influence of Rectangular Prism Pien Error on Angle Measurement for Photoelectric Autocollimator. Proceedings of the 4th International Conference on Instrumentation and Measurement, Computer, Communication and Control (IMCCC), Harbin Inst Technol.

[224] Yang Z., Fan B., Xi Q., Wang C., Wang R. (2018). Research on the Method of Building Space Rectangular Coordinate System of Photoelectric Autocollimator. Acta Metrol. Sin..

[225] Liu Y., Zhen Y., Xie L., Wang W., Yang J., Li R. (2022). Roll angle of autocollimator measurement method based on hollow cube corner reflector. Optoelectron. Lett..

[226] Konyakhin I.A., Turgalieva T.V. (2013). Three-coordinate digital autocollimator. J. Opt. Technol..

[227] Shur V.L., Lukin A.Y., Shestopalov Y.N., Popov O.I. (2005). Two-coordinate digital autocollimator. Meas. Tech..

[228] Zou Y., Zhang N., Yuan H., Liu C., Liu D. (2022). High precision deflection angle measurement of irregular light spot. Chin. Space Sci. Technol..

[229] Guo Y., Cheng H., Liu G. (2023). Three-degree-of-freedom autocollimation angle measurement method based on crosshair displacement and rotation. Rev. Sci. Instrum..

[230] Yan L., Yan Y., Chen B., Lou Y. (2022). A Differential Phase-Modulated Interferometer with Rotational Error Compensation for Precision Displacement Measurement. Appl. Sci..

[231] Cai Y., Yang B., Fan K.-C. (2019). Robust roll angular error measurement system for precision machines. Opt. Express.

[232] Ren W., Cui J., Tan J. (2022). Precision roll angle measurement system based on autocollimation. Appl. Opt..

[233] Schwenke H., Neuschaefer-Rube U., Pfeifer T., Kunzmann H. (2002). Optical methods for dimensional metrology in production engineering. CIRP Ann.-Manuf. Technol..

[234] Zhang C., Liu W., Duan F., Fu X., Li X., Yan M. (2022). A novel method for reducing the defocus error of a five-degree-of-freedom measurement system. Proc. SPIE.

[235] Qibo F., Bin Z., Cunxing C., Cuifang K., Yusheng Z., Fenglin Y. (2013). Development of a simple system for simultaneously measuring 6DOF geometric motion errors of a linear guide. Opt. Express.

[236] Cui C., Feng Q., Zhang B., Zhao Y. (2016). System for simultaneously measuring 6DOF geometric motion errors using a polarization maintaining fiber-coupled dual-frequency laser. Opt. Express.

[237] Cai Y., Lou Z., Ling S., Liao B.-s., Fan K.-c. (2018). Development of a Compact Three-Degree-of-Freedom Laser Measurement System with Self-Wavelength Correction for Displacement Feedback of a Nanopositioning Stage. Appl. Sci..

[238] Wenzheng L., Cong Z., Fajie D., Xiao F., Ruijia B., Xiuming L., Ming Y. (2022). A high-precision calibration method of PSD in long-distance laser auto-collimation system. Proc. SPIE.

[239] Li B., Dong W., Xiao A., Yu M., Ge W., He J. (2021). High-precision non-contact two-dimensional dynamic angle measurement system based on PSD. Laser Infrared.

[240] Du M.-x., Yan Y.-f., Zhang R., Cai C.-l., Yu X., Bai S.-p., Yu Y. (2022). 3D position angle measurement based on a lens array. Chin. Opt..

[241] Li R., Zhen Y., Di K., Wang W., Nikitin M., Tong M.H., Zhang Y., Zou X., Konyakhin I. (2021). Three-degree-of-freedom autocollimator with large angle-measurement range. Meas. Sci. Technol..

[242] Gu W., Zhang X., Liu J., Zheng J., Huang H., Jia L., Qu X., Zhang F. (2023). Optical Angle Barcodes Enabled by a Pixelated Metasurface for Absolute Micro-Angle Measurement. Laser Photonics Rev..

[243] Cai C., Yan Y., Zhang L., Du M., Yu X. (2022). A Lens-Array-Based Measurement Technique for Spatial Angle. IEEE Access.

[244] Barboza R., Babazadeh A., Marrucci L., Cardano F., de Lisio C., D’Ambrosio V. (2022). Ultra-sensitive measurement of transverse displacements with linear photonic gears. Nat. Commun..

[245] Yuan G.H., Zheludev N.I. (2019). Detecting nanometric displacements with optical ruler metrology. Science.

[246] Zang H.F., Xi Z., Zhang Z.Y., Lu Y.H., Wang P. (2022). Ultrasensitive and long-range transverse displacement metrology with polarization-encoded metasurface. Sci. Adv..

[247] Li R., Xiao H., Xie L., Feng T., Ma Y., Guo J., Zhou M., Nikitan M., Konyakhin I. (2022). Autocollimation angle-measurement method with a large range based on spot deformation. Opt. Express.

[248] Geckeler R.D., Schumann M., Just A., Krause M., Lassila A., Heikkinen V. (2022). A comparison of traceable spatial angle autocollimator calibrations performed by PTB and VTT MIKES. Metrologia.

[249] Kranz O., Geckeler R.D., Just A., Krause M., Osten W. (2015). From plane to spatial angles: PTB’s spatial angle autocollimator calibrator. Adv. Opt. Technol..

[250] Watanabe T., Fujimoto H., Masuda T. (2005). Self-calibratable rotary encoder. J. Phys. Conf. Ser..

[251] Stone J.A., Amer M., Faust B., Zimmerman J. (2003). Angle metrology using AAMACS and two small-angle measurement systems. Dev. Traceable Dimens. Meas. II.

[252] Burnashev M.N., Pavlov P.A., Filatov Y.V. (2013). Development of precision laser goniometer systems. Quantum Electron..

[253] Shi J., Li Y.C., Tao Z.X., Zhang D.X., Xing H.Y., Tan J.B. (2022). High-precision autocollimation method based on a multiscale convolution neural network for angle measurement. Opt. Express.

